# The *Chlamydia trachomatis* type III secretion substrates CT142, CT143, and CT144 are secreted into the lumen of the inclusion

**DOI:** 10.1371/journal.pone.0178856

**Published:** 2017-06-16

**Authors:** Maria da Cunha, Sara V. Pais, Joana N. Bugalhão, Luís Jaime Mota

**Affiliations:** 1 UCIBIO—REQUIMTE, Departamento de Ciências da Vida, Faculdade de Ciências e Tecnologia, Universidade Nova de Lisboa, Caparica, Portugal; 2 Instituto de Tecnologia Química e Biológica António Xavier, Universidade Nova de Lisboa, Oeiras, Portugal; University of the Pacific, UNITED STATES

## Abstract

*Chlamydia trachomatis* is a human bacterial pathogen causing ocular and genital infections. It multiplies exclusively within an intracellular membrane-bound vacuole, the inclusion, and uses a type III secretion system to manipulate host cells by injecting them with bacterially-encoded effector proteins. In this work, we characterized the expression and subcellular localization in infected host cells of the *C*. *trachomatis* CT142, CT143, and CT144 proteins, which we previously showed to be type III secretion substrates. Transcriptional analyses in *C*. *trachomatis* confirmed the prediction that *ct142*, *ct143* and *ct144* are organized in an operon and revealed that their expression is likely driven by the main σ factor, σ^66^. In host cells infected by *C*. *trachomatis*, production of CT142 and CT143 could be detected by immunoblotting from 20–26 h post-infection. Immunofluorescence microscopy of infected cells revealed that from 20 h post-infection CT143 appeared mostly as globular structures outside of the bacterial cells but within the lumen of the inclusion. Furthermore, immunofluorescence microscopy of cells infected by *C*. *trachomatis* strains carrying plasmids producing CT142, CT143, or CT144 under the control of the *ct142* promoter and with a C-terminal double hemagglutinin (2HA) epitope tag revealed that CT142-2HA, CT143-2HA or CT144-2HA showed an identical localization to chromosomally-encoded CT143. Moreover, CT142-2HA or CT144-2HA and CT143 produced by the same bacteria co-localized in the lumen of the inclusion. Overall, these data suggest that the CT142, CT143, and CT144 type III secretion substrates are secreted into the lumen of the inclusion where they might form a protein complex.

## Introduction

*Chlamydiae* are a large group of obligate intracellular bacteria including nine families [1]: *Chlamydiaceae*, which comprises a single genus (*Chlamydia*) grouping bacterial species that are pathogens of humans and other vertebrates; and 8 families of microorganisms that infect a variety of vertebrate and non-vertebrate animals as well as free-living amoebae [2]. All *Chlamydiae* undergo a developmental cycle involving the inter-conversion between a non-replicative infectious form, the elementary body (EB), and a replicative non-infectious form, the reticulate body (RB) [3]. Intracellular multiplication of *Chlamydiae* occurs exclusively within a membranous vacuolar compartment known as inclusion. Among *Chlamydiaceae*, *C*. *trachomatis* serovars, which are divided in ocular, urogenital and lymphogranuloma venereum (LGV) strains, are the leading cause of infectious blindness in developing countries [4] and the most prevalent sexually transmitted bacteria worldwide [5].

Type III secretion (T3S) systems are present in many Gram-negative bacteria establishing pathogenic or symbiotic relationships with their hosts. They mediate the delivery of bacterially-encoded effector proteins into eukaryotic host cells [6, 7]. *Chlamydiae* use a T3S system throughout the developmental cycle to transport several effectors across the host cell plasma membrane and the inclusion membrane [3, 8]. Some of these proteins, such as TarP [9], CT694 [10], or TepP [11], are packed in EBs and are delivered into host cells during the early phases of infection to mediate invasion, by modulation of the host actin cytoskeleton [12, 13], and to subvert immune signaling [11, 14]. Another class of chlamydial T3S effectors, the inclusion membrane (Inc) proteins [15, 16], insert into the inclusion membrane and interfere with several host cell processes such as cytoskeleton dynamics [17, 18], vesicular and non-vesicular transport [19, 20], death [21], or immune signalling [22]. Other *Chlamydia* T3S effectors have been identified and shown to interfere, e.g., with host cell ubiquitination [23], histone methylation [24], or the ESCRT machinery [25]. In addition, *C*. *trachomatis* T3S substrates that are enzymes for glycogen metabolism have been shown to localize in the lumen of the inclusion [26].

Several *C*. *trachomatis* T3S effectors have been identified but it is likely that others remain to be characterized. Before methods for transformation and regulated ectopic expression of proteins in *C*. *trachomatis* have been described [27–29], a main methodology to search and identify putative chlamydial T3S effectors relied on using heterologous host bacteria, such as *Salmonella* [30], *Shigella* [23, 24, 31, 32], or *Yersinia* [9, 10, 33–37], carrying well characterized T3S systems. While many candidate T3S substrates identified in these studies have been validated in cells infected by *C*. *trachomatis* (e.g. Inc proteins, CopN, TarP, CT694, CT620, CT621, CT711, or CT737/NUE), many remain to be characterized in further detail. Using *Y*. *enterocolitica* as a heterologous host, we previously described the identification of 10 likely *C*. *trachomatis* candidate T3S substrates (CT053, CT105, CT142, CT143, CT144, CT161, CT338, CT429, CT656, and CT849) [37]. Among the genes encoding these proteins, the expression of *ct142*, *ct143* and *ct144* has been shown to be distinctly up-regulated by a protein (Pgp4) encoded in the *Chlamydia* virulence plasmid [38, 39]. This plasmid is present in many chlamydial species [39], and studies in animal models of infection showed it is a virulence factor *in vivo* [40–42].

In this work, we characterized the genetic organization of *ct142*, *ct143* and *ct144* in the *C*. *trachomatis* chromosome and showed that during infection of host cells the encoding proteins (CT142, CT143, and CT144) localize within the inclusion in globular structures that appear outside of the bacterial cells. These observations support the concept that *C*. *trachomatis* T3S substrates can be secreted into the lumen of the inclusion.

## Materials and methods

### Cell lines

HeLa 229 and Vero cells (from the European Collection of Cell Culture; ECACC) were maintained in high-glucose Dulbecco’s modified Eagle Medium (DMEM; Thermo Fisher Scientific) supplemented with heat-inactivated 10% (v/v) fetal bovine serum (FBS; Thermo Fisher Scientific) at 37°C in a humidified atmosphere of 5% (v/v) CO_2_.

### Bacterial strains and growth conditions

*Escherichia coli* TOP10 (Thermo Fisher Scientific) was used for construction and purification of plasmids, *E*. *coli* BL21 (DE3) (Novagen) for recombinant protein expression, and *E*. *coli* ER2925 (New England Biolabs) to amplify and purify plasmids for transformation of *C*. *trachomatis*. *E*. *coli* strains were routinely grown in liquid or solid lysogeny broth (LB) medium with the appropriate antibiotics and supplements. Plasmids were introduced into *E*. *coli* by electroporation.

*C*. *trachomatis* LGV serovar L2 strains 434/Bu (L2/434; from ATCC) and 25667R (L2/2566R; [43]; kindly provided by Agathe Subtil) were propagated in HeLa 229 cells using standard techniques [44]. Throughout this work we used the nomenclature of the annotated *C*. *trachomatis* D/UW3 strain [45]. Transformation of *C*. *trachomatis*, first reported by the laboratory of Ian N. Clarke [27], was done essentially as described by Agaisse and Derré [28]. The optimal penicillin G concentration to select transformants was 1 U/ml. Once established, the transformed strains were cultured in the presence of 10 U/ml of penicillin and plaque purified using Vero cells, as described by Nguyen and Valdivia [46].

### DNA manipulation, plasmids and primers

The plasmids used in this work, their main characteristics and construction details, are described in S1 Table. The DNA primers used in their construction are listed in S2 Table. Plasmids were constructed and purified using standard molecular biology procedures with proof-reading Phusion DNA polymerase (Thermo Fisher Scientific), restriction enzymes (Thermo Fisher Scientific), T4 DNA Ligase (Thermo Fisher Scientific), DreamTaq DNA polymerase (Thermo Fisher Scientific), DNA clean & concentrator^TM^-5 kit and Zymoclean^TM^ gel DNA recovery kit (Zymo Research), and GeneElute Plasmid Miniprep kit (Sigma Aldrich) or NZYMidiprep kit (NZYtech), according to the instructions of the manufacturers. The backbone plasmids used in this work were pGEX-4T-2 (GE Healthcare) and pMal-c (New England Biolabs), for recombinant protein purification, and pEGFP-C1 (Clontech) for transfection of mammalian cells. Furthermore, pSVP247 (S1 Fig and S1 Table), a derivative of p2TK2-SW2 [28], was the backbone to generate *C*. *trachomatis* expression plasmids bearing genes whose transcription is halted by the *incD* terminator (T_*incD*_) and encoding proteins with a double hemagglutinin epitope tag (2HA) at their C-terminus. The accuracy of the nucleotide sequence of all the inserts in the constructed plasmids was confirmed by DNA sequencing.

### Infection of HeLa 229 cells with *C*. *trachomatis*

For immunofluorescence analysis, 5x10^4^ HeLa 229 cells were seeded per well in 24 well plates containing 13 mm glass coverslips. For immunoblotting 1x10^5^ HeLa 229 cells were seeded per well in 24 well plates. Scaling-up was done as appropriate for other cell culture plates or flasks. The day after seeding, media was replaced by Hank's balanced salt solution (HBSS) and the cells were incubated ∼15 min at 37°C in a humidified atmosphere of 5% [v/v] CO_2_, while the *C*. *trachomatis* inocula (previously titrated infectious particles, as described by Scidmore [44]) were prepared in either sucrose phosphate glutamate buffer (SPG; 0.2 mM sucrose, 17 mM Na_2_HPO_4_, 3 mM NaH_2_PO_4_, 5 mM L-glutamic acid) or HBSS. The buffer was then removed and the *C*. *trachomatis* inocula were added at a multiplicity of infection (MOI) of 1–5 and incubated for 30 min at 37°C in a humidified atmosphere of 5% [v/v] CO_2_ (24 or 6 well-plates) or at 60 min at room temperature with gentle rocking (tissue culture flasks). At this point, the inocula were removed and replaced by DMEM supplemented with 10% (v/v) FBS and 10 μg/ml of gentamicin. This was considered the time zero of infection.

To quantify infectious progeny, HeLa cells infected by *C*. *trachomatis* strains for different times were lysed by osmotic shock (15 min incubation in sterile H_2_O). Dilutions of these lysates in HBSS were used to infect freshly seeded HeLa cells. The newly infected cells were fixed after 22–26 h, *C*. *trachomatis* bacteria were immunolabelled, and the number of inclusion forming units (IFUs) was calculated as described by Scidmore [44].

### Transcription linkage analysis

HeLa cells were infected with *C*. *trachomatis* L2/434 for 20 h and RNA was isolated using NZY total RNA kit (NZYTech). cDNA was obtained by reverse transcription PCR (RT-PCR) using random hexamers and iSCRIPT (Bio-Rad). The product of a typical reverse transcription reaction but without iSCRIPT (cDNA^-^) and *C*. *trachomatis* genomic DNA (gDNA) isolated using NZY tissue gDNA kit (NZYTech) were also used as template in control RT-PCR reactions. Specific primers-pair combinations (S2 Table) were designed in order to determine possible transcriptional linkages.

### 5´rapid amplification of cDNA ends (RACE)

The identification of the transcription start site of *ct142* in *C*. *trachomatis* L2/434 was done using a 5´/3´RACE kit (second generation; Roche). RNA was isolated from HeLa cells infected for 42 h by *C*. *trachomatis* L2/434 and RNA was isolated as described above for the transcription linkage analysis. The primers complementary to *ct142* that were used are listed in S2 Table. Final PCR amplification of double stranded cDNA was done with Phusion DNA polymerase (Thermo Fisher Scientific). PCR products were purified after agarose gel electrophoresis, using High Pure PCR purification kit (Roche), and then subjected to DNA sequencing. All these manipulations were done according to instructions from the manufacturers.

### Recombinant protein expression and purification

*E*. *coli* BL21(DE3) carrying a pGEX-4T-2-derived plasmid (encoding CT143 with GST fused at its N-terminus; GST-CT143) or pMal-c derived plasmids (encoding MBP, or CT142 or CT143 with MBP fused at their N-termini; MBP-CT142, MBP-CT143) were used for recombinant protein expression by auto-induction [47]. In both cases, bacterial cultures were grown for 4 h at 37°C, with an agitation of 150 rotations per minute (rpm), and then shifted to 25°C for an additional 24 h, with an agitation of 150 rpm. The bacterial cells were harvested by centrifugation at 10,500 x *g* for 15 min at 4°C.

The pellet of cells expressing GST-CT143 was resuspended in ice-cold phosphate-buffered saline (PBS) containing 1% [v/v] Triton X-100, 10 mM dithiothreitol (DTT), lyzozyme (10 mg/ml), benzonase® (Novagen), and a protease inhibitor cocktail (Amresco). The cells were lysed using BugBuster® (Novagen) according to the instructions of the manufacturer. The lysates were centrifuged at 10,500 x *g* for 30 min at 4°C, after which the supernatants were loaded onto equilibrated gluthathione sepharose beads (GE Healthcare) pre-packed in empty 1 ml columns (MoBiTec). The column was washed 3 times with 5 column volumes of ice-cold binding buffer (PBS containing 0.05% [v/v] Triton X-100 and 1 mM DTT). After the washing step, GST-CT143 was eluted with ice-cold Tris-HCl, pH 8.0, containing 10 mM gluthathione.

Pellets of cells expressing MBP, MBP-CT142 or MBP-CT143 were resuspended in ice-cold column buffer (200 mM NaCl, 20 mM Tris-HCl [pH 7.4], 1 mM ethylenediaminetetraacetic acid [EDTA], 1 mM DTT). The cells were lysed by passing twice through a French Press (18,000 lb/in^2^) in the presence of 1 mM phenylmethylsulfonyl fluoride (PMSF). The lysates were centrifuged at 10,500 x *g* for 30 min at 4°C, after which the supernatants were loaded onto equilibrated amylose resin (New England Biolabs), according to the instructions of the manufacturer. The column was washed 12 times with 1 ml of ice-cold column buffer, after which the proteins were eluted with ice-cold column buffer containing 10 mM maltose.

Purified proteins were dialysed using SnakeSkin Dialysis Tubing^TM^ (Thermo Fisher Scientific) according to the instruction of the manufacturer.

### Antibodies

Polyclonal antibodies against CT142 were generated against three synthetic peptides conjugated to ovalbumin: peptide 1 (36–50 amino acid residues) NH_2_-VKSISAKESFSVKRKC-COOH; peptide 2 (260–274 amino acid residues) NH_2_-CKGGDYVDKSALSTLY-COOH; peptide 3 (126–140 amino acid residues) NH_2_-QKLPLIGPSRLVYQSC-COOH (Metabion, Germany). The resulting antibodies were affinity purified on CNBr-sepharose matrix containing the respective peptides (Metabion).

Purified GST-CT143 was used to immunize New Zealand White rabbits for the production of polyclonal anti-CT143 sera (Davids Biotechnologie, Germany). For purification, the anti-CT143 serum was firstly incubated with acetone powders obtained from *E*. *coli* BL21 (DE3) containing pGEX-4T-2, allowing the serum to be partly depleted of the anti-GST antibodies or any contaminant *E*. *coli* protein in purified GST-CT143. Secondly, anti-CT143 serum was affinity purified on 1 mg of MBP-CT143 immobilized on a nitrocellulose membrane. The affinity-purified antibodies were concentrated using Amicon Ultra-4 (Millipore) and quantified using NanoDrop 1000 (Thermo Fisher Scientific).

The following primary antibodies were used: rabbit polyclonal anti-CT142 (this work; 1:200 dilution for immunoblotting); rabbit polyclonal anti-CT143 (this work; 1:200 dilution for immunoblotting and 1:50 dilution for immunofluorescence); mouse monoclonal anti-chlamydial Hsp60 (A57-B9; Thermo Fisher Scientific; 1:1,000 dilution for immunoblotting and 1:200 dilution for immunofluorescence); goat polyclonal anti-MOMP of *C*. *trachomatis* (Abcam; 1:1,000 dilution for immunoblotting and 1:500 dilution for immunofluorescence); goat anti-*Chlamydia trachomatis* fluorescein isothiocyanate (FITC)-conjugated polyclonal antibody (Millipore; 1:100 dilution for immunofluorescence); rat monoclonal anti-HA (3F10; Roche; 1:1,000 dilution for immunoblotting and 1:200 dilution for immunofluorescence), mouse monoclonal anti-tubulin (clone B-5-1-2; Sigma Aldrich; 1:1,000 for immunoblotting).

For immunoblotting, the secondary antibodies used were all horseradish peroxidase (HRP)-conjugated (GE Healthcare and Jackson ImmunoResearch; used at 1:10,000). For immunofluorescence, the following secondary antibodies were used: cyanine 5 (Cy5)-conjugated donkey anti-goat (Jackson ImmunoResearch Laboratories; 1:200), Alexa Fluor®-594 (AF594)-conjugated AffiniPure donkey anti-goat (Jackson ImmunoResearch Laboratories; 1:200); Rhodamine Red-X-conjugated AffiniPure donkey anti-rabbit (Jackson ImmunoResearch Laboratories; 1:200); AF488-conjugated goat anti-mouse (Jackson ImmunoResearch Laboratories; 1:200); Rhodamine Red-X-conjugated anti-rat (Jackson ImmunoResearch Laboratories; 1:200); and AF488-conjugated AffiniPure Donkey anti-rat (Jackson ImmunoResearch Laboratories; 1:200).

### Immunoblotting

To harvest infected HeLa cells, the cells were washed once with PBS and then detached with 50 μl (per well in a 24 well plate) of TrypLE^TM^ Express (Life Technologies) by incubation during 5 min at 37°C. The cells were then collected, pelleted by a brief centrifugation, washed 2 times with ice-cold PBS, and stored at -80°C until use. Prior to SDS-PAGE, the cell pellets were thawed, resuspended in an appropriate volume of SDS-PAGE loading buffer and the proteins were further denatured by an incubation of 5 minutes at 100°C, followed by incubation with benzonase (Novagen). Samples were separated by 12% (v/v) SDS-PAGE and transferred to 0.2 μm nitrocellulose membranes (Bio-Rad) using Trans-Blot Turbo Transfer System (BioRad). Immunoblot detection was done with SuperSignal® West Pico Chemiluminescent Substrate (Thermo Fisher Scientific) or SuperSignal® West Femto Maximum Sensitivity Substrate (Thermo Fisher Scientific) and exposure to Amersham Hyperfilm ECL (GE Healthcare). Quantification of bands in immunoblots was performed by densitometry analysis using Fiji software [48].

### Immunofluorescence microscopy

Infected HeLa cells were fixed in either PBS containing 4% (w/v) paraformaldehyde (PFA) for 15 min or in methanol (-20°C) for 10 min. For immunostaining, the antibodies were diluted in PBS containing 10% [v/v] horse serum (when fixation was done with PFA, 0.1% [v/v] Triton X-100 was added to allow permeabilization of cells). After immunolabelling, the cells were consecutively washed with PBS and H_2_O. The coverslips were assembled using Aqua-poly/Mount (Polysciences) on microscopy glass slides, and the cells were examined by conventional fluorescence microscopy or by confocal microscopy. Images were processed and assembled using Adobe Photoshop. Quantitative analyses of immunofluorescence images were performed using Fiji software [48].

## Results

### *C*. *trachomatis ct142*, c*t143*, and *ct144* are organized in an operon

**Fig 1:**
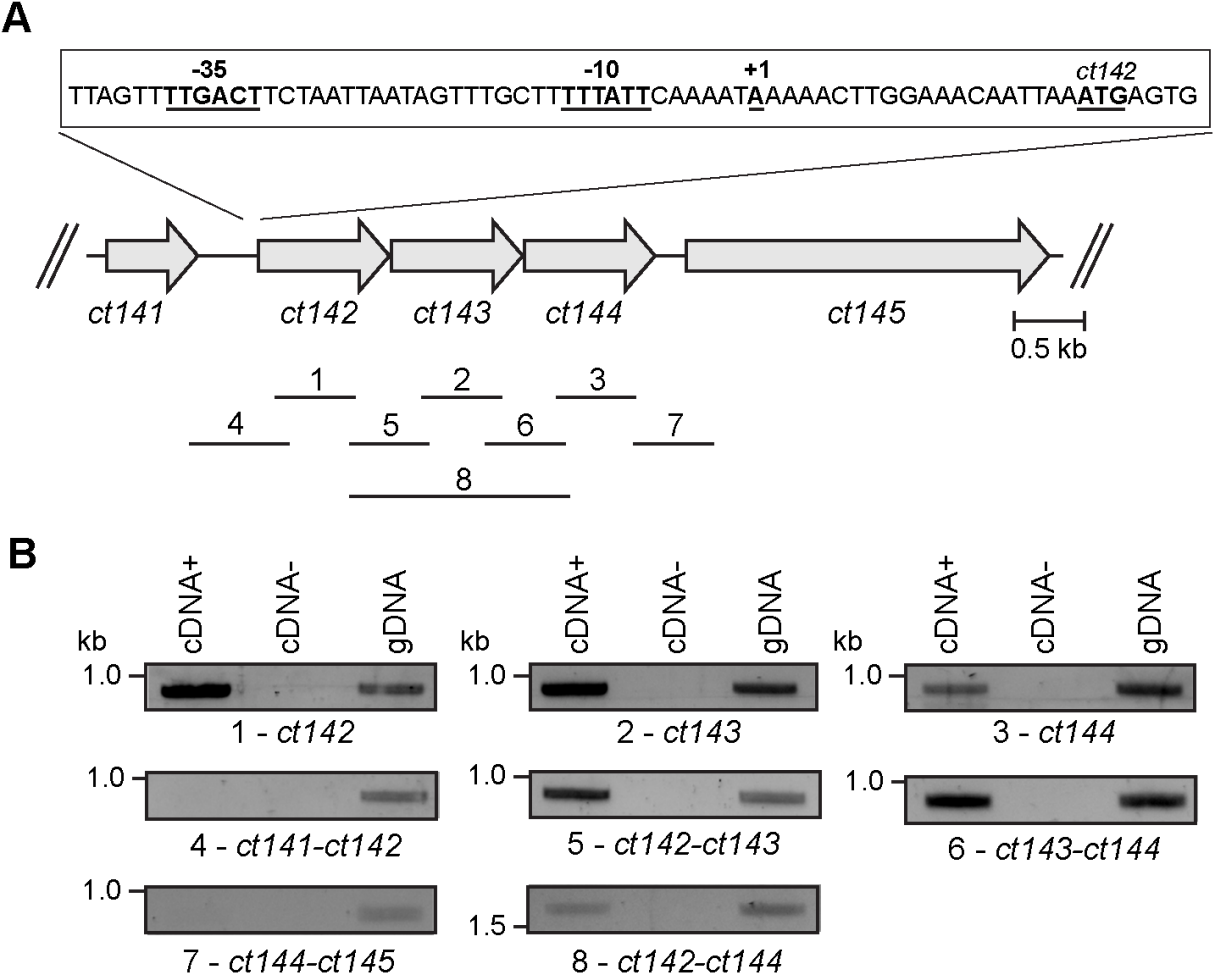
Characterization of *ct142*, *ct143*, and *ct144*. (A) Genetic organization of *ct142*, *ct143* and *ct144* in the chromosome of *C*. *trachomatis* depicting the fragments (1–8) amplified in the transcription linkage analyses, the transcriptional start site determined by 5´RACE as well as the deduced -10 and -35 σ^66^-like promoter regions. (B) Transcription linkage analyses in *C*. *trachomatis* L2/434: cDNA+, PCR from cDNA generated with reverse transcriptase (RT) from bacterial RNA isolated from infected HeLa cells; cDNA-, as cDNA+ but without RT; gDNA, PCR from bacterial DNA.

The *ct142*, *ct143*, and *ct144* genes (named *ctl0397*, *ctl0398*, and *ctl0399*, respectively, in strain L2/434) are localized adjacently in the chromosome of *C*. *trachomatis* (Fig 1A). The CT142, CT143 and CT144 proteins are highly conserved in all *C*. *trachomatis* serovars (> 95% of amino acid identity for CT142; > 96% of amino acid identity for CT143; and > 90% of amino acid identity for CT144) and are also conserved in *Chlamydia* (S3 Table). However, a PSI-BLAST analysis [49] failed to identify any significant amino acid sequence similarity with proteins from the other *Chlamydiae* families (S3 Table). The *ct142*, *ct143* and *ct144* genes are syntenic in the chromosome of the majority of the *Chlamydia* spp. (S2 Fig). In *C*. *pneumoniae* and *C*. *felis* the genes are located in the complementary strand, and in *C*. *pneumoniae* the orthologue of *ct143* is duplicated (S3 Table and S2 Fig).

Previous studies revealed similar mRNA levels and an identical profile of expression of *ct142*, *ct143* and *ct144* during the developmental cycle of *C*. *trachomatis* [37]. These observations and the genetic organization of *ct142*, *ct143* and *ct144* suggested that these genes could be organized in an operon. To examine this, RNA was isolated from HeLa cells infected by *C*. *trachomatis* L2/434 for 20 h and cDNA was generated by RT-PCR. Specific primer pairs (S2 Table) were then used in conventional PCR reactions to determine possible transcriptional linkages between *ct142*, *ct143*, and *ct144* (Fig 1A) and genes upstream from *ct142* (*ct141*) and downstream from *ct144* (*ct145*). This showed that *ct142*, *ct143* and *ct144* are transcriptionally linked (Fig 1B), indicating that they are organized in an operon. There was no detectable transcriptional linkage between *ct141* and *ct142* or between *ct144* and *ct145* (Fig 1B). To define the promoter of *ct142*, we determined its transcription start site by 5´RACE using as template RNA isolated from HeLa cells infected by *C*. *trachomatis* L2/434 for 42 h, and primers complementary to *ct142* (S2 Table). By inspecting the nucleotide sequences immediately upstream from the determined transcription start site of *ct142* (Fig 1A) for sequences that could be recognized by *C*. *trachomatis* σ factors, σ^66^ (the homolog of *E*. *coli* main σ factor, σ^70^), σ^54^ (an alternative σ factor) and σ^28^ (a minor σ factor) [50], we only identified -10 and -35 regions possibly recognized by σ^66^ (Fig 1A).

Overall, these experiments showed that *ct142*, *ct143*, and *ct144* are organized in an operon whose transcription is directed from a σ^66^-like promoter (*ct142* promoter; P_*ct142*_) about 30 nucleotides upstream from the start codon of *ct142*.

### CT142 and CT143 are produced during the developmental cycle of *C*. *trachomatis*

**Fig 2:**
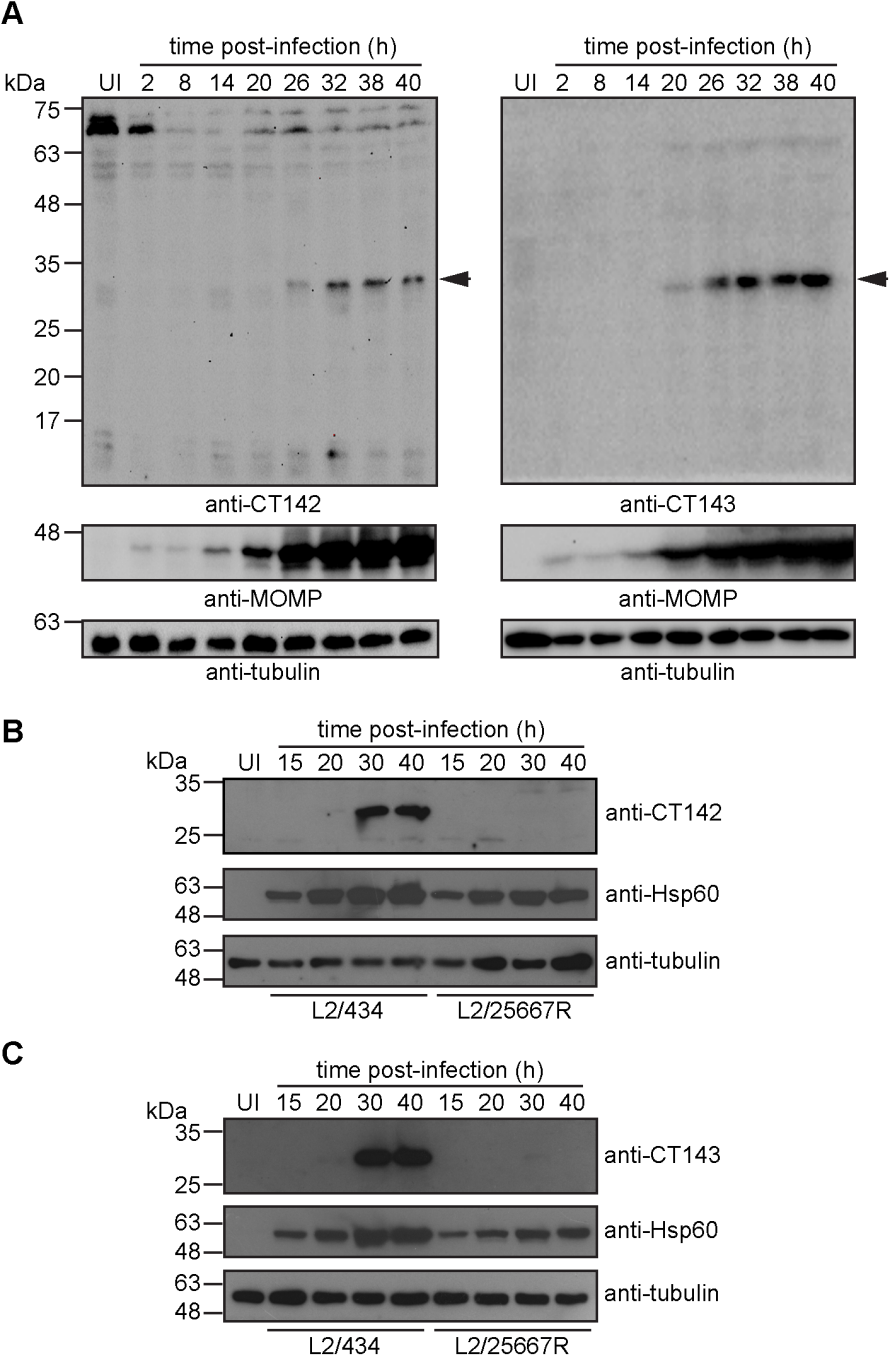
Production of CT142 and CT143 by *C*. *trachomatis* L2/434. (A) HeLa cells were either left uninfected (UI) or infected by *C*. *trachomatis* L2/434 for 2, 8, 14, 20, 26, 32, 38 or 40 h. Whole cell lysates were analyzed by immunoblotting with antibodies against CT142 (blot on the left) or CT143 (blot on the right), *C*. *trachomatis* major outer membrane protein (MOMP; bacterial loading control) and tubulin (loading control for host cells). The arrows indicate the position of CT142 and CT143 bands. (B) and (C) HeLa cells were either left uninfected (UI) or infected by *C*. *trachomatis* L2/434 or L2/25667 for 15, 20, 30 or 40 h, as indicated. Whole cell lysates were analyzed by immunoblotting with antibodies against CT142 (B) or CT143 (C), *C*. *trachomatis* Hsp60 (bacterial loading control) and tubulin (loading control for host cells).

To analyze the production of CT142, CT143, and CT144 by *C*. *trachomatis* during the bacterial developmental cycle, we aimed to obtain antibodies against these proteins. We generated antibodies that specifically recognized CT142 and CT143 by immunoblotting when the proteins were ectopically expressed in HeLa cells (S3 Fig), but we did not succeed in generating antibodies recognizing CT144. We then analyzed by immunoblotting the presence of CT142 and CT143 in extracts of HeLa cells either uninfected or infected by *C*. *trachomatis* L2/434 for 2, 8, 14, 20, 26, 32, 38, or 40 h. Bands that migrated on SDS-PAGE according to the predicted molecular mass of CT142 (31.5 kDa) and CT143 (31 kDa) could be detected from 20 h post-infection (p.i.), and more obviously from 26 h p.i. (Fig 2A). At 20 h p.i., the immunoblot signal detected by each antibody was very weak and in some experiments not even visible. Transcription of *ct142* and *ct143* (and also of *ct144*) is up-regulated by *C*. *trachomatis* plasmid-encoded Pgp4 [38]. Therefore, to further ascertain the specificity of the anti-CT142 and anti-CT143 antibodies, we analyzed by immunoblotting extracts of HeLa cells infected for 15, 20, 30 or 40 h by *C*. *trachomatis* L2/434 or L2/25667R (lacking the *C*. *trachomatis* virulence plasmid). In extracts from cells infected by *C*. *trachomatis* L2/434, bands migrating as proteins of ∼31 kDa were again detected with the anti-CT142 (Fig 2B) or anti-CT143 antibodies (Fig 2C). However, in extracts of HeLa cells infected by *C*. *trachomatis* L2/25667R no clear bands migrating as proteins of ∼31 kDa could be detected with any of the antibodies (Fig 2B and 2C).

In summary, these experiments confirmed that CT142 and CT143 are produced during the developmental cycle of *C*. *trachomatis* and further illustrated that their expression is dependent on the *Chlamydia* plasmid.

### CT143 localizes within the *C*. *trachomatis* inclusion but outside of the bacterial cells

**Fig 3:**
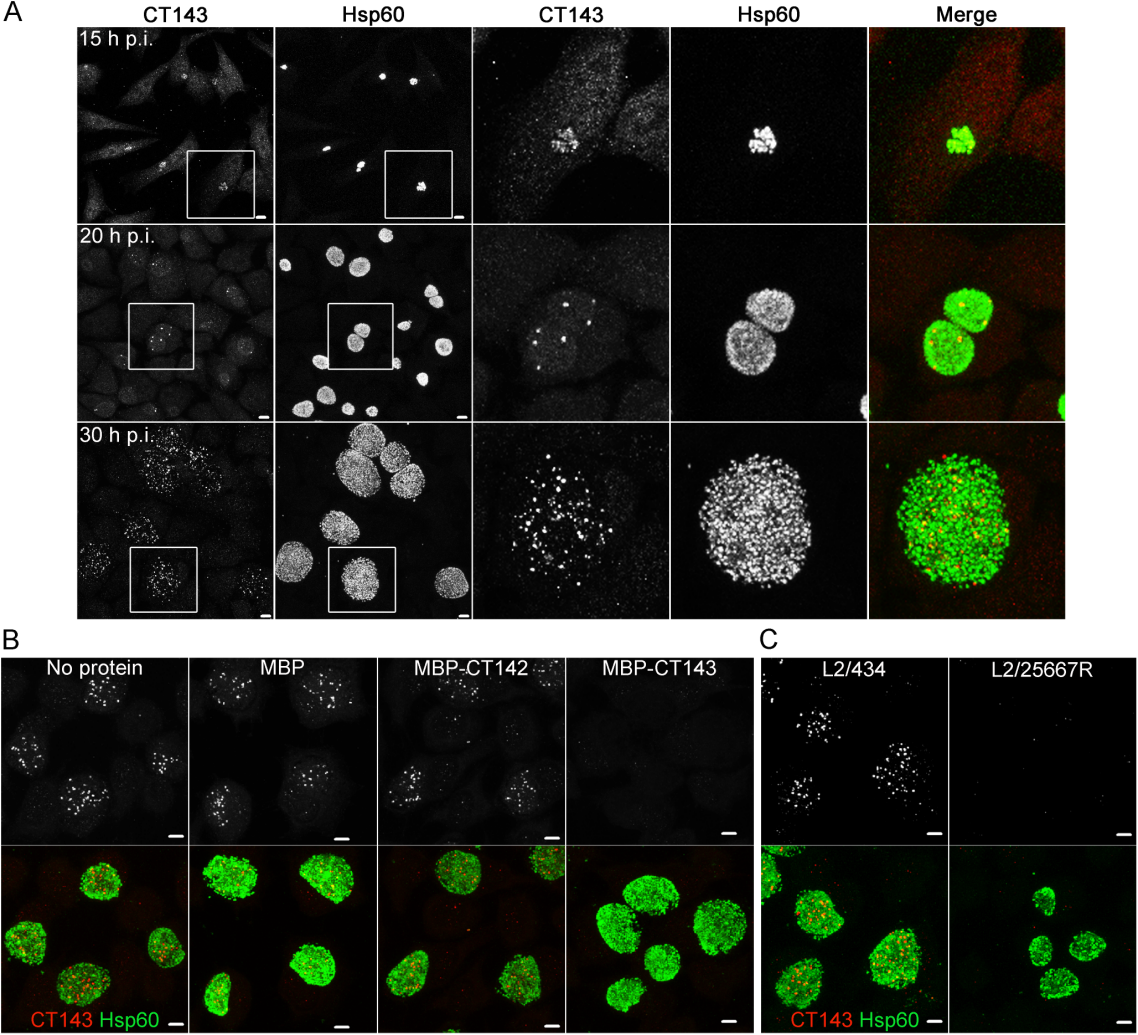
Specific immunolabelling of CT143 in HeLa cells infected by *C*. *trachomatis*. HeLa cells were infected by *C*. *trachomatis* L2/434 for 15, 20 and 30 h (A), for 30 h (B), or by L2/434 or *C*. *trachomatis* L2/25667R for 30 h (C). In all cases, cells were fixed with methanol, immunolabelled with anti-CT143 and anti-Hsp60 and adequate fluorophore-conjugated secondary antibodies, and analyzed by confocal immunofluorescence microscopy. In (B), before immunolabelling, anti-CT143 antibodies were incubated in the presence of 8 μg of purified MBP, MBP-CT142 or MBP-CT143, as indicated. All images are combined projections of multiple 0.2 μm *z*-sections. In (A), images were zoomed 3-fold in the area delimited by a white box. All scale bars, 5 μm.

We previously showed that CT142, CT143, and CT144 are T3S substrates [37]. Therefore, using the anti-CT142 and anti-CT143 antibodies, we aimed to detect the presence of these proteins in the cytoplasm of infected host cells. However, the analysis could only be done for CT143 because the anti-CT142 antibody did not generate an immunofluorescence-specific signal. For this, HeLa cells were infected by *C*. *trachomatis* L2/434 for 15, 20, or 30 h. At these time-points, the cells were fixed with methanol, immunolabelled for CT143 and Hsp60 (a *Chlamydia* cytosolic molecular chaperone), and then analyzed by indirect immunofluorescence confocal microscopy. At 15 h p.i., the immunofluorescence signal for CT143 was weak and overlapped with the Hsp60 signal (Fig 3A). At 20 h p.i., the immunofluorescence signal for CT143 was more intense but still restricted to the inclusion (Fig 3A). However, the signal did not appear to overlap with the Hsp60 signal and instead we observed discrete globular structures (Fig 3A). At 30 h p.i., these anti-CT143-labelled structures were much more abundant (Fig 3A), and they were distinct from the Hsp60 signal and restricted to the inclusion (Fig 3A).

The observations described above suggested that in infected cells the majority of CT143 localizes within the inclusion but apparently outside of the bacterial cell. To ensure that the observed anti-CT143 immunofluorescence signal was specific, prior to immunolabelling of HeLa cells infected for 30 h with the L2/434 strain, the anti-CT143 antibody was incubated in the presence of an excess of purified MBP, MBP-CT142 or MBP-CT143. As control, the anti-CT143 antibody underwent a similar incubation but without any added protein. This showed that only the pre-incubation with MBP-CT143, but not with MBP or MBP-CT142, led to a clear reduction of the anti-CT143 immunofluorescence signal (Fig 3B). In addition, we compared the anti-CT143 immunofluorescence signal in HeLa cells infected for 30 h by *C*. *trachomatis* strains L2/434 or plasmidless L2/25667R. This revealed that the CT143 signal was drastically reduced in cells infected by the L2/25667R strain (Fig 3C). Overall, this indicated that the immunofluorescence signal detected with the anti-CT143 antibody was specific.

**Fig 4:**
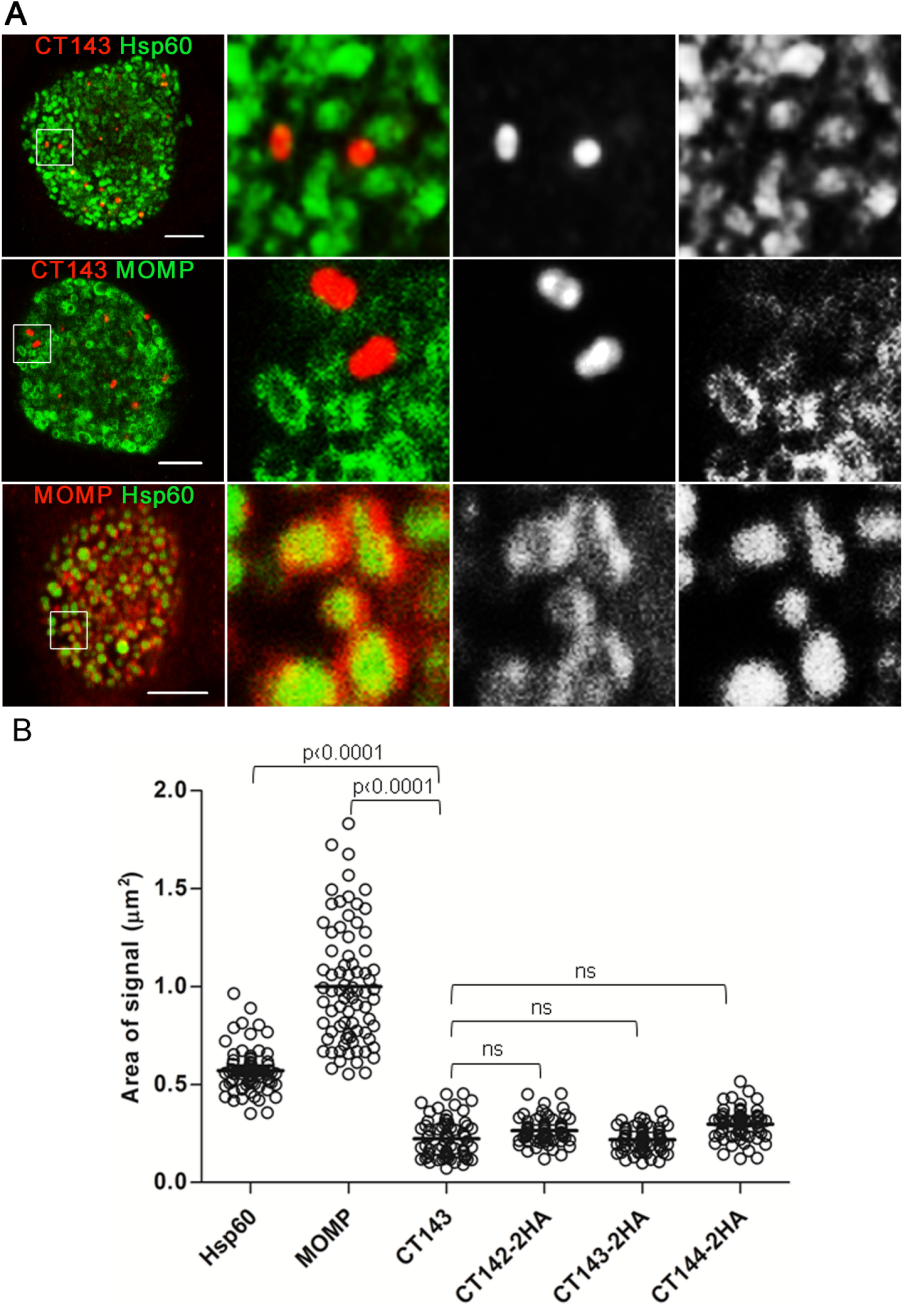
CT143 localizes within in the *C*. *trachomatis* inclusion but outside of the bacterial cells. (A) HeLa 229 cells were infected with *C*. *trachomatis* L2/434 for 30 h, fixed with methanol, and immunolabelled for CT143 and anti-Hsp60 (upper panel), CT143 and MOMP (middle panel), or MOMP and Hsp60 (lower panel), and adequate fluorophore-conjugated secondary antibodies. Cells were then analyzed by confocal immunofluorescence microscopy. Images correspond to single *z* sections. The images were zoomed 6-fold in the area delimited by a white box. Scale bars, 5 μm. (B) Comparison of the area of the immunofluorescence signal of Hsp60, MOMP, CT143, CT142-2HA, CT143-2HA and CT144-2HA (see Fig 4). Measurements were done using Fiji software for at least 90 particles randomly chosen from independent images. P-values were calculated by a two-tailed unpaired Student’s *t*-test and considered significant if P < 0.05.

To evaluate in more detail the subcellular localization of CT143, HeLa cells infected by strain L2/434 for 30 h were immunolabelled for CT143 and Hsp60 (as before), for CT143 and *C*. *trachomatis* major outer membrane protein (MOMP), and for Hsp60 and MOMP (Fig 4A). This confirmed that CT143 appeared as intra-inclusion globular structures, as the anti-CT143 immunofluorescence signal did not co-localize with the bacterial signal from the anti-MOMP or anti-Hsp60 antibodies (Fig 4A; upper and middle panels). In contrast, and as expected, the anti-MOMP signal (bacterial outer membrane) mostly encircled the anti-Hsp60 signal (bacterial cytoplasm) (Fig 4A; lower panel). We then measured the area of each of the immunofluorescence signals analyzed (CT143, Hsp60 and MOMP). This revealed that the area of the anti-CT143 signal is considerably smaller than the area of the anti-Hsp60 or anti-MOMP signals (Fig 4B).

Overall, these results indicated that, from about 20 h p.i., CT143 localizes within the inclusion but outside of the bacterial cells. Therefore, CT143 is likely secreted into the lumen of the inclusion.

### The chlamydial developmental cycle is not significantly affected in *C*. *trachomatis* strains over-producing CT142-2HA, CT143-2HA, CT144-2HA, or CT142, CT143, and CT144-2HA

**Fig 5:**
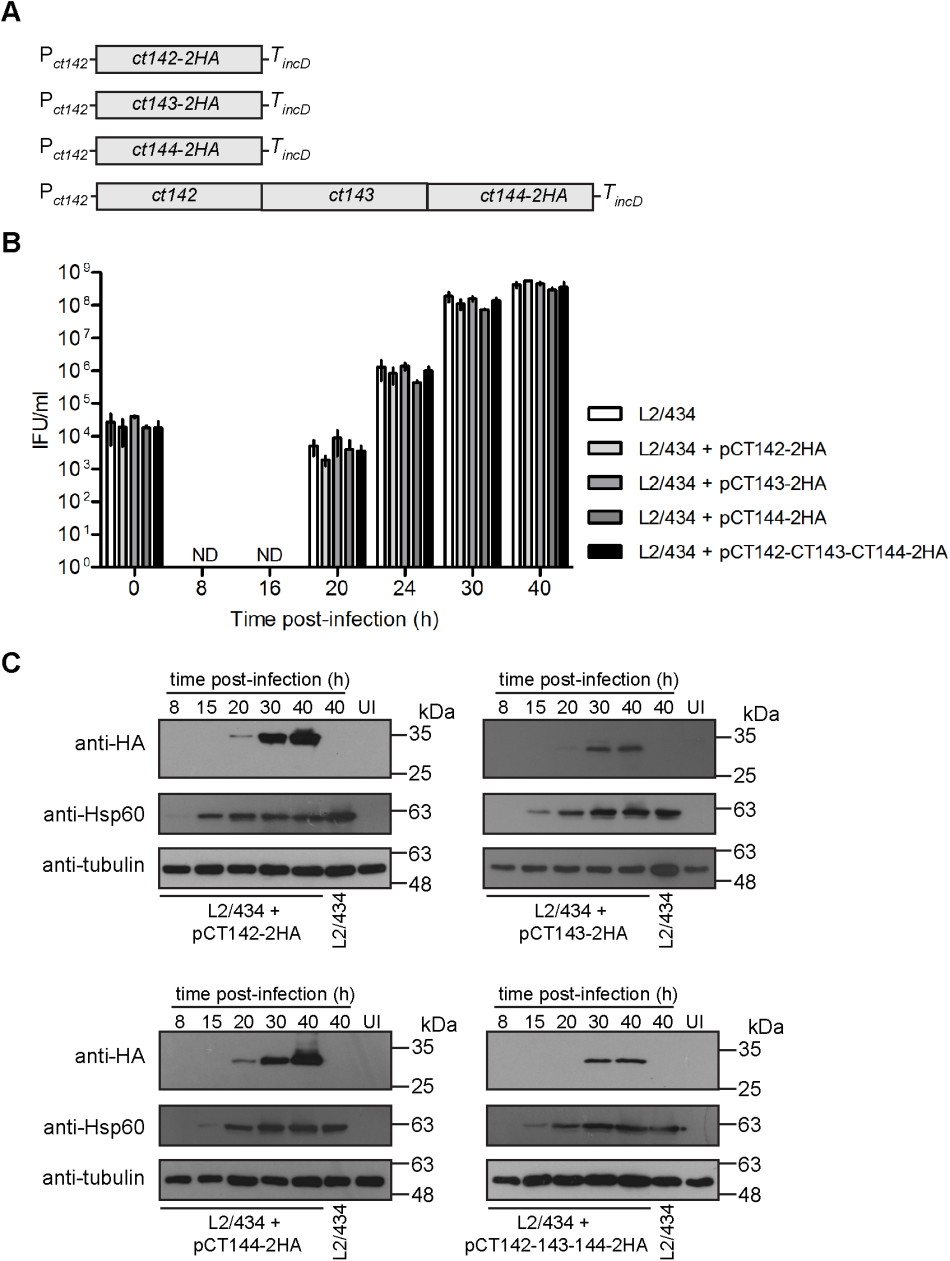
Generation and characterization of *C*. *trachomatis* strains producing CT142-2HA, CT143-2HA and/or CT144-2HA. (A) Schematic representation of the genes present in the plasmid of each recombinant *C*. *trachomatis* strain. P_*ct142*_, *ct142* promoter; T_*incD*_, *incD* terminator. (B) HeLa cells were infected with the indicated strains at a multiplicity of infection of 5 and recoverable inclusion forming units (IFUs) were determined at 0, 8, 16, 20, 24, 30, and 40 h p.i. Data are mean and standard error of the mean of 3 independent experiments. (C) HeLa cells were either left uninfected (UI) or infected by the indicated *C*. *trachomatis* strains for 8, 15, 20, 30, and 40 h. Whole cell lysates were analyzed by immunoblotting with antibodies against HA, *C*. *trachomatis* Hsp60 (bacterial loading control) and tubulin (loading control for host cells).

To further validate the observations regarding the localization of CT143 and to analyze the localization in infected cells of other proteins (CT142 and CT144) encoded in the *ct142-ct143-ct144* operon, we transformed *C*. *trachomatis* L2/434 with four different p2TK2-SW2-derived plasmids [28] encoding CT142-2HA (pCT142-2HA), CT143-2HA (pCT143-2HA), CT144-2HA (pCT144-2HA), or CT142, CT143 and CT144-2HA (pCT142-CT143-CT144-2HA) (Fig 5A). In all plasmids, expression of the genes encoding these proteins is driven by P_*ct142*_ (DNA sequence shown in Fig 1A plus additional nucleotides upstream, in a total of 165 nucleotides upstream from the start codon of *ct142*) and halted by the terminator of the *C*. *trachomatis incD* gene (T_*incD*_) (Fig 5A).

To test if the constructed *C*. *trachomatis* strains were affected in their developmental cycle, we quantified the number of infectious progeny at different times of infection of HeLa cells by comparison to the parental L2/434 strain. This revealed that the kinetics of appearance of infectious particles during the developmental cycle of the four newly constructed strains was similar to the one of L2/434 (Fig 5B), indicating that the overall bacterial physiology was not affected by the plasmids or by overexpression of CT142, CT143, and/or CT144 proteins. We also followed the production of CT142-2HA, CT143-2HA, and CT144-2HA by immunoblotting of extracts of HeLa cells infected for 8, 15, 20, 30 or 40 h with each of the four *C*. *trachomatis* strains expressing these proteins. As for endogenous CT142 and CT143 (Fig 2B and 2C), expression of CT142-2HA, CT143-2HA and CT144-2HA could be detected from 20 h p.i. but the protein levels were much higher at 30 or 40 h p.i. (Fig 5C). For CT144-2HA encoded by plasmid pCT142-CT143-CT144-2HA, expression of CT144-2HA was only detected at 30 h p.i. (Fig 5C). We also quantified the levels of CT142-2HA, CT143-2HA and CT144-2HA produced by the four strains at 30 h p.i. While there were apparently higher levels of CT142-2HA and of CT144-2HA (encoded by pCT144-2HA) relative to CT143-2HA and CT144-2HA (encoded by pCT142-CT143-CT144-2HA), the differences were not statistically significant (Fig A and Fig B in S4 Fig). Finally, the levels of CT142-2HA or of CT143-2HA were ∼9-fold higher (9 ± 1 for CT142-2HA, and 9 ± 3 for CT143-2HA) than the levels of endogenous CT142 or CT143 in strain L2/434 (Fig A, Fig C, and Fig D in S4 Fig). Production of plasmid-encoded CT142-2HA or CT144-2HA led to an apparent increase in production of chromosomally-encoded CT142 and CT143, but the observed differences were not statistically significant (Fig A, Fig C, and Fig D in S4 Fig).

In summary, the developmental cycle of the constructed *C*. *trachomatis* strains (Fig 5A) is not significantly different from that of the parental strain and, as expected by the predicted ∼8 copies of the *C*. *trachomatis* virulence plasmid [51], plasmid-encoded CT142-2HA and CT143-2HA proteins are produced at higher levels than chromosomally-encoded CT142 and CT143.

### Plasmid-encoded CT142-2HA, CT143-2HA and CT144-2HA also localize within the inclusion but outside of the bacteria

**Fig 6:**
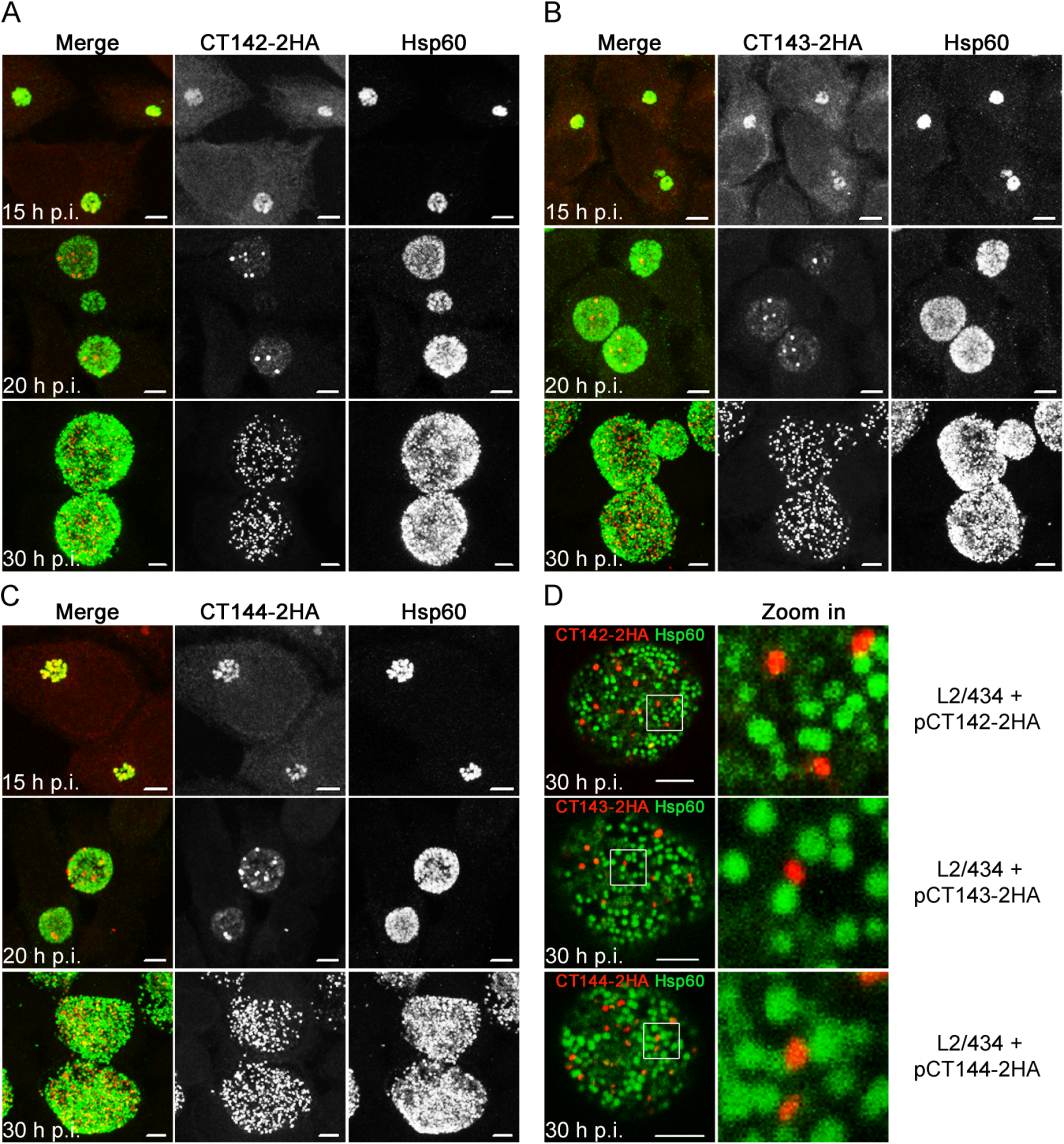
CT142-HA, CT143-2HA, and CT144-2HA localize within the *C*. *trachomatis* inclusion but outside of the bacteria. HeLa cells were infected for 15, 20 or 30 h with *C*. *trachomatis* L2/434 harbouring pCT142-2HA (A), pCT143-2HA (B), or pCT144-2HA (C). The infected cells were fixed with methanol, immunolabelled with anti-HA and anti-Hsp60 antibodies, and appropriate fluorophore-conjugated secondary antibodies, and analyzed by confocal immunofluorescence microscopy. Images are combined projections of multiple 0.2 μm *z*-sections. Scale bars, 5 μm. (D) HeLa cells infected for 30 h with the indicated *C*. *trachomatis* strains were fixed, immunolabelled and analyzed by confocal immunofluorescence microscopy, as indicated in (A), (B), and (C). Images are single *z* sections. In the area delimited by a white square, images were zoomed 5-fold. Scale bars, 5 μm.

To analyze the localization of plasmid-encoded CT142-2HA, CT143-2HA, and CT144-2HA, we infected HeLa cells with *C*. *trachomatis* L2/434-derived strains bearing pCT142-2HA, pCT143-2HA or pCT144-2HA. The cells were fixed at 15, 20, and 30 h p.i., immunolabelled for HA and for Hsp60 and then analyzed by indirect immunofluorescence confocal microscopy. Overall, the observations made for the plasmid-encoded proteins were very similar to what was observed for chromosomally-encoded CT143 in cells fixed at the same times p.i. (compare Fig 6A–6C with Fig 3A): at 15 h p.i., for CT142-2HA, CT143-2HA or CT144-2HA, we detected a weak HA signal that overlapped with the Hsp60 signal (Fig 6A–6C); at 20 h p.i., the HA signal of CT142-2HA (Fig 6A), CT143-2HA (Fig 6B) or CT144-2HA (Fig 6C) appeared as inclusion-restricted discrete globular structures that did not seem to overlap with the Hsp60 signal (Fig 6A–6C); at 30 h p.i., these structures were much more abundant, did not seem to co-localize with the Hsp60 signal, and were restricted to the inclusion (Fig 6A–6C).

We then analyzed in more detail the HA immunofluorescence signal observed at 30 h p.i. in HeLa cells infected by the *C*. *trachomatis* strains producing CT142-2HA, CT143-2HA, or CT144-2HA (Fig 6D). This confirmed that the globular structures revealed by the HA signal of each of the proteins did not overlap with the Hsp60 signal (Fig 6D). Additionally, the area defined by the HA signal for each protein encoded by pCT142-2HA, pCT143-2HA or pCT144-2HA was significantly smaller than the area defined by the Hsp60 immunofluorescence signal (Fig 4B), but identical to the area defined by the CT143 immunofluorescence signal in cells infected by *C*. *trachomatis* L2/434 (Fig 4B).

In summary, as for chromosomally-encoded CT143, plasmid-encoded and over-expressed CT142-2HA, CT143-2HA, and CT144-2HA localize in the inclusion but outside of the bacteria, suggesting that from about 20 h p.i. all three proteins are secreted into the lumen of the inclusion.

### CT143 co-localizes with CT142-2HA and CT144-2HA in the lumen of the inclusion

**Fig 7:**
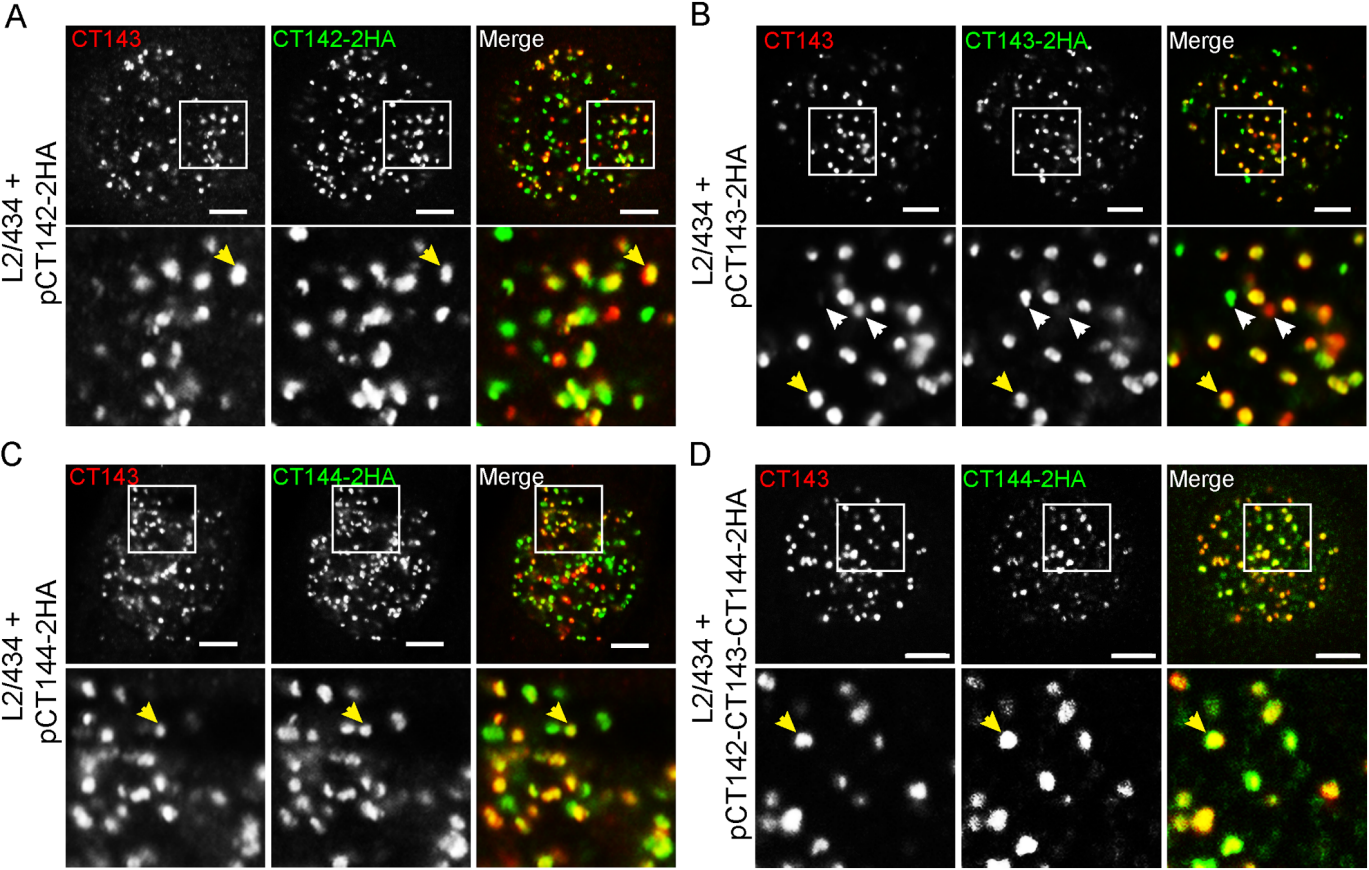
Co-localization between intra-inclusion structures revealed by CT143 and CT142-2HA, CT143-2HA or CT144-2HA. HeLa 229 cells were infected for 30 h by *C*. *trachomatis* L2/434 bearing pCT142-2HA (A), pCT143-2HA (B), pCT144-2HA (C), or pCT142-CT143-CT144-2HA (D). The cells fixed with methanol and immunolabelled with anti-CT143 and anti-HA antibodies and appropriate fluorophore-conjugated antibodies. Stained cells were analyzed by confocal immunofluorescence microscopy. Images are single *z* sections. Yellow arrows exemplify co-localizing globular structures and white arrows highlight globular structures not showing obvious co-localization. In the area delimited by a white square (upper panels) images were zoomed 3-fold (lower panels). All scale bars, 5 μm.

We next asked if the anti-CT143 immunofluorescence signal would co-localize with the anti-HA immunofluorescence signal of CT142-2HA or CT144-2HA. As control, we also analyzed the co-localization between the anti-CT143 and anti-HA immunofluorescence signals of CT143 and CT143-2HA, respectively. For this, HeLa cells were infected for 30 h by *C*. *trachomatis* L2/434-derived strains bearing pCT142-2HA, pCT143-2HA, pCT144-2HA, or pCT142-143-144-2HA. The infected cells were then fixed and immunolabelled using anti-CT143 and anti-HA antibodies. Analysis by immunofluorescence confocal microscopy of the intra-inclusion globular structures characteristic of CT143, CT142-2HA, CT143-2HA or CT144-2HA revealed co-localization between CT143 and CT142-2HA (Fig 7A), CT143 and CT143-2HA, as expected, (Fig 7B), and CT143 and CT144-2HA (Fig 7C and 7D). This suggested that CT142, CT143, and CT144 could be part of protein complexes within the lumen of the inclusion. In all cases analyzed, even for CT143 relative to CT143-2HA, the co-localization was obvious but not perfect (Fig 7). It is possible that some epitopes might be inaccessible within the putative protein complexes. Moreover, the protein levels of chromosomally-encoded CT143 relative to plasmid-encoded proteins could have interfered with the degree of co-localization observed.

In summary, although the observed co-localization between CT143 and CT142-2HA or CT144-2HA does not indicate that the proteins interact, these data suggested that the globular structures revealed by immunolabelling of CT143, CT142-2HA, CT143-2HA or CT144-2HA might correspond to multi-protein complexes within the lumen of the inclusion.

## Discussion

We found that in infected host cells the *C*. *trachomatis* T3S substrates CT142, CT143, and CT144 localize in apparently large globular structures within the inclusion but outside of the bacteria. This suggests that these proteins are secreted into the lumen of the inclusion. It remains possible that CT142, CT143, and CT144 might be delivered into the cytoplasm of host cells in levels too low to be detectable by immunofluorescence microscopy. However, our observations do indicate that the major proportion of these proteins is translocated across the two bacterial membranes into the lumen of the inclusion. We made identical observations for chromosomally-encoded CT143 (using an anti-CT143 antibody) and for ∼9-fold over-expressed, plasmid-encoded and epitope-tagged CT143-2HA. Therefore, by analogy, the presence of CT142-2HA and CT144-2HA in the lumen of the inclusion should also reflect the localization of endogenous CT142 and CT144.

It is generally believed that T3S systems are mostly used to transport bacterially encoded proteins directly into the cytoplasm of eukaryotic host cells, after protein translocation across the bacterial and host cell membranes. However, our observations on the localization of CT142, CT143, and CT144 in infected cells suggest that the *C*. *trachomatis* T3S system also mediates the transport of proteins into the lumen of the inclusion, after protein translocation only across the bacterial membranes. There are however at least two other possible scenarios explaining the predominant localization of CT142, CT143, and CT144 within the inclusion lumen. One is that, although we have shown that these proteins are T3S substrates using *Y*. *enterocolitica* as a heterologous host [37], they are in fact secreted into the lumen of the *C*. *trachomatis* inclusion by another pathway. However, using another bacteria as heterologous host to study T3S signals in chlamydial proteins has been commonly used in the field [10, 31, 33, 52] and is normally accepted as valid. A way to further test if CT142, CT143, and CT144 are T3S substrates during the developmental cycle of *C*. *trachomatis* could be to use small molecules (salicylidene acylhydrazides) that have been described to function as inhibitors of T3S systems [53] and to disrupt the progression of the *Chlamydia* developmental cycle [54]. However, the specificity of these small molecules against the T3S system is very unclear [55–59] and therefore this approach would not be the most appropriate. A T3S system-deficient *C*. *trachomatis* mutant would be required to test without any doubt if CT142, CT143, and CT144 are type III secreted in infected cells, but such mutant would likely be non-viable because of the expected essential role of the T3S system in entry of *Chlamydia* into host cells and subsequent intracellular development [3, 8]. In the future, it might be possible to generate a *C*. *trachomatis* conditional mutant deficient in the T3S system, but this is much beyond the scope of this work. A second possibility is that the proteins are delivered into the host cell cytoplasm by the *C*. *trachomatis* T3S system and then translocated back across the vacuolar membrane into the inclusion. This seems very unlikely because it would be a rather complex transport pathway and we never detected even residual specific immunostaining of CT142-2HA, CT143 or CT143-2HA, or CT144-2HA in the host cytoplasm.

Based on the previous discussion and on observations from other studies, we do favor the possibility that the *C*. *trachomatis* T3S system can transport proteins into the lumen of the inclusion [7]. First, there are precedents for *C*. *trachomatis* T3S substrates found in that localization. The glycogen metabolizing enzymes (GlgA, GlgB, GlgX, GlgP, and MalQ) have been shown to be T3S substrates using *Shigella flexneri* as heterologous host, and GlgA and GlgX have been immunolocalized in the lumen of the inclusion [26, 60]. GlgA was also detected in the host cytoplasm [60], and GlgX was also found in the inclusion membrane [26]. Other examples are CT620 and CT621, which were identified as T3S substrates using *S*. *flexneri* as heterologous host and found in the lumen of the inclusion as well as in the cytoplasm of host cells [32]. *C*. *trachomatis* Pls1/CT049 and Pls2/CT050 were also described to be secreted into the lumen of the inclusion [61], and also appear as globular structures that are reminiscent of those related to CT142, CT143 and CT144, but they might not be T3S substrates [61]. Second, there is no conceptual reason for why a system capable of transporting substrates across the bacterial membranes and a host cell membrane would not, in some conditions, also translocate proteins only across the bacterial membranes. In fact, even if this might be only an experimental artifact, many bacteria possessing T3S systems can be induced to secrete protein substrates in the extracellular medium [62, 63], and in *Yersinia* it has been proposed that different signals direct T3S substrates into distinct locations [64]. In the case of *C*. *trachomatis*, we speculate that T3S substrates can gain direct access to the cytoplasm of host cells (e.g., TarP, Incs), be released in the lumen of the inclusion and also into the cytosol of host cells (CT620, CT621, GlgA), or simply secreted into the lumen of the inclusion (CT142, CT143, CT144 and GlgX), from where they might also reach the inclusion membrane (GlgX). Evidently, this hypothetical scenario would imply complex regulatory mechanisms of protein transport that remain to be elucidated.

Chromosomally-encoded CT143 co-localize with plasmid-encoded CT142-2HA or CT144-2HA in the globular structures within the inclusion, which suggests that CT142, CT143, and CT144 could be part of a protein complex. However, thus far, we have been unable to detect specific interactions between these proteins within infected host cells. At the present, the function of a putative protein complex including CT142, CT143 and CT144 in the lumen of inclusion is elusive. Given the marked up-regulation of expression of the *ct142*, *ct143*, and *ct144* genes by Pgp4 [38], the function of CT142, CT143, and CT144 could be related to activities that have been attributed to the *Chlamydia* virulence plasmid [39]. In particular, lytic exit of *C*. *trachomatis* from host cells is dependent on the plasmid-encoded transcriptional regulator Pgp4 and can be inhibited by a compound that has been shown to block the T3S system [65]. It is however unclear how CT142, CT143, and CT144 could influence bacterial exit from their localization within the lumen of the inclusion. One can also speculate if upon host cell exit of the bacteria the putative CT142-C1143-CT144 complex might function as decoy for the immune system. Plasmid-encoded Pgp4 also controls several chromosomal genes encoding proteins involved in glycogen synthesis [38, 66]. The activity of these proteins leads to the characteristic accumulation of glycogen granules within the inclusion [26, 67]. It is therefore also possible that the putative CT142-CT143-CT144 complex might be related to the glycogen granules and that the activity of CT142, CT143, and CT144 could be related to glycogen metabolism. To address whether CT142, CT143, and CT144 are involved in these processes, or in other possible hypotheses concerning their function, future studies should be directed at using recently described methods [68–70] to inactivate the *ct142*, *ct143*, or *ct144* genes. Furthermore, proteomic analysis of the putative CT142-CT143-CT144 complex isolated from infected cells might identify other *C*. *trachomatis* proteins associating with it.

We also clarified that the genes encoding CT142, CT143, and CT144 form an operon and their expression is likely driven by a σ^66^ promoter upstream from the start codon of *ct142*. In a previous RNA sequencing analysis of the transcriptome of *C*. *trachomatis* LGV serovar L2b strain UCH-1/proctitis there was no obvious definition of a transcription start site for *ct142* (*CTLon_0393*), *ct143* (*CTLon_0394*), or *ct144* (*CTLon_0395*) [71]. It remains possible that in addition to the identified P_*ct142*_, transcription of *ct142*, *ct143*, or *ct144* could be directed by other promoters at different times or conditions of the *C*. *trachomatis* developmental cycle. However, this seems unlikely as we detected no significant differences between the 3 genes in a previous real-time quantitative PCR analysis of their mRNA levels during the developmental cycle of strain L2/434 [37].

In summary, this work contributed for the characterization of *C*. *trachomatis* T3S substrates and, in line with a recent study [26], further suggests that the chlamydial T3S system could also mediate protein secretion into the lumen of the inclusion. Additional studies are needed to solidify this intriguing hypothesis and to understand the putative regulatory processes controlling protein transport by the same machinery into the lumen of the inclusion and into and across the vacuolar membrane. The function of CT142, CT143, and CT144 also remains to be defined. This should be directly analyzed using recently developed methods for genetic manipulation of *C*. *trachomatis* [68–70].

## Supporting information

S1 Table Plasmids used in this work. (PDF)

S2 Table DNA primers used in this work. (PDF)

S3 Table Identification of orthologues of *C*. *trachomatis* CT142 (CTL0397), CT143 (CTL0398), and CT144 (CTL0399) in other *Chlamydiae*. (PDF)

S1 Fig Map of plasmid pSVP247. Details of plasmid construction are in S1 Table. The pSW2 plasmid backbone [27] is shown in black, the *Escherichia coli* origin of replication (*ori*) in yellow, the ampicillin resistance gene (bla) in blue, the multiple cloning site (MCS) in grey, and the double hemagglutinin (2HA) epitope tag and *incD* terminator in different tones of red. The DNA sequence of the MCS with unique restriction sites and the 2HA-encoding region are depicted in the box below the plasmid map.
(PDF)

S2 Fig Genetic organization of *ct142*, *ct143* and *ct144* orthologues in *Chlamydiaceae*. Orthologues of *ct142* are depicted in green, orthologues of *ct143* are depicted in red, and orthologues of *ct144* are depicted in pink. The syntenic organization of the three genes is illustrated in *C*. *trachomatis* serovar A (strain A/HAR-13), *C*. *trachomatis* serovar B (strain B/Jali20/OT), *C*. *trachomatis* serovar D (strain D/UW-3/CX) as well as in *C*. *muridarum* Nigg, *C*. *abortus* S26/3, *C*. *caviae* GPIC, *C*. *felis* Fe/C-56 and *C*. *pneumoniae* CWLO29. The initial image was obtained from the ChlamydiaeDB.org website (http://liferay.csb.univie.ac.at/portal/web/chlamydiaedb) and then adapted.
(PDF)

S3 Fig Characterization of antibodies against CT142 and CT143. HeLa cells were either left untransfected (NT) or transfected using jetPEI® (Polyplus-transfection) with plasmids encoding EGFP, EGFP-CT143 or EGFP-CT142, as indicated. Whole cell lysates were analyzed with antibodies against GFP, CT142 or CT143. Bands corresponding to EGFP-CT142 and EGFP-CT143 are indicated by an arrow.
(PDF)

S4 Fig Quantification of CT142, CT143, and CT144 proteins produced by *C*. *trachomatis* recombinant strains. (A) HeLa cells were left uninfected (UI) or infected for 30 h by *C*. *trachomatis* L2/434 or L2/434 carrying plasmids pCT142-2HA, pCT143-2HA, pCT144-2HA or pCT142-CT143-CT144-2HA. Whole cell lysates were analyzed by immunoblotting with antibodies against HA, CT142 or CT143 (as indicated), *C*. *trachomatis* Hsp60 (bacterial loading control) and tubulin (loading control for host cells). The arrows indicate the position of CT142, CT142-2HA, CT143 and CT143-2HA bands. (B) The bands corresponding to HA-tagged proteins in each lane of the anti-HA blot in Fig A in S4 Fig (and in other replicates) were quantified by densitometry relative to the corresponding bands of Hsp60 and tubulin using Fiji software [48]. The calculated CT142/Hsp60/tubulin values in the graph indicate mean ± standard error of the mean (SEM) from 4 independent experiments. P-values were calculated by a one way ANOVA and Tukey post hoc analysis and the values were not significantly different (P > 0.05) between the different data sets. (C and D) The bands corresponding to CT142 and CT142-2HA proteins (C), or the bands corresponding to CT143 and CT143-2HA proteins (D), in each lane of the anti-CT142 or CT143 blots, respectively, in Fig A in S4 Fig (and in other replicates) were quantified by densitometry relative to the corresponding bands of Hsp60 and tubulin using Fiji software [48]. The calculated HA/Hsp60/tubulin values in the graph indicate mean ± SEM from 4 independent experiments, relative to the values of CT142 (in C) or CT143 (in D) in cells infected by L2/434. P-values were calculated by a one way ANOVA and Dunett post hoc analysis (relative to the L2/434 data) and there were significant differences (P < 0.05) only for data corresponding to samples infected by L2/434 harbouring pCT142-2HA or pCT142-CT143-CT144-2HA (in C) or for data corresponding to samples infected by L2/434 harbouring pCT143-2HA.
(PDF)
