# Supplementary material for: The Chlamydia trachomatis type III secretion substrates CT142, CT143, and CT144 are secreted into the lumen of the inclusion
Source: PLoS One. 2017 Jun 16;12(6):e0178856. doi: 10.1371/journal.pone.0178856 (PMC5473537; doi:10.1371/journal.pone.0178856)
Supplement: S1 Table — (PDF) [file pone.0178856.s001.pdf]

S1 Table. Plasmids used in this work.

**Name**	**Description/Construction**[a]	**Source/Reference**
pGEX-4T-2	*Escherichia coli* expression vector to generate fusions to the C- terminus of glutathione S-transferase (GST) (Amp^R).	GE Healthcare
pMal-c	*E. coli* expression vector to generate fusions to the C-terminus of maltose-binding protein (MBP) (Amp^R).	New England Biolabs
p2TK2-SW2	*E. coli* and *Chlamydia trachomatis* shuttle vector (Amp^R).	Isabelle Derré [28]
pBAD/*Myc*-His A	*E. coli* expression vector (Amp^R).	Thermo Fisher Scientific
pEGFP-C1	Mammalian transfection vector to generate fusions to the C-terminus of EGFP (Km^R).	Clontech
pSC1	Derivative of pBAD/*Myc*-His A encoding TarP with a double hemagglutinin epitope tag (2HA) at its C-terminus. *tarP* was amplified by PCR from DNA of the *C. trachomatis* serovar L2 434/Bu (L2/434) strain using primers #5 and #6 (Table S2). The DNA product was digested with BspHI and HindIII and ligated into pBAD/*Myc*-His A digested with NcoI and HindIII.	This work
pSVP247	Derivative of p2TK2-SW2 enabling the construction of plasmids encoding proteins with a 2xHA at their C-terminus and with the *incD* transcription terminator (T_{incD}) sequence (Fig. S1). The DNA sequence encoding 2HA was amplified by PCR from pSC1 using primers #1486 and #1488 (Table S2). The DNA sequence of T_{incD} was amplified by PCR from DNA of *C. trachomatis* L2/434 using primers #1487 and #1483 (Table S2). The two products were fused by overlapping PCR using primers #1486 and #1483 (Table S2); the resulting DNA product was digested with NotI and SalI and ligated into those sites of p2TK2-SW2.	This work

S1 Table. ***Continued.***

Name	Description/Construction[a]	Source/Reference
pCM33	Derivative of pMal-c encoding MBP-CT142. *ct142* was amplified from genomic DNA of the *C. trachomatis* L2/434 strain using primers #418 and #490 (Table S2). The DNA product was digested with HindIII and BamHI and ligated into those sites of pMal-c.	This work
pMC47	Derivative of pGEX-4T-2 encoding GST-CT143. *ct143* was amplified from genomic DNA of *C. trachomatis* L2/434 strain using primers #417 and #491 (Table S2). The DNA product was digested with XhoI and EcoRI and ligated into those sites of pGEX-4T-2.	This work
pMC56	Derivative of pMal-c encoding MBP-CT143. *ct143* was amplified from DNA of *C. trachomatis* L2/434 strain using primers #1050 and #1051 (Table S2). The DNA product was digested with EcoRI and PstI and ligated into those sites of pMal-c.	This work
pMC70 (pCT142-2HA)	Derivative of pSVP247 encoding CT142-2HA expressed under the control of the *ct142* promoter. *ct142* and its promoter region were amplified by PCR from DNA of *C. trachomatis* L2/434 using primers #1652 and #1555 (Table S2). The product was digested with KpnI and NotI and ligated into those sites of pSVP247.	This work
pMC71 (pCT143-2HA)	Derivative of pSVP247 encoding CT143-2HA expressed under the control of the *ct142* promoter. The promoter region of *ct142* and the *ct143* gene were amplified by PCR from DNA of *C. trachomatis* L2/434 using primers #1652 and #1653 (Table S2), and #1654 and #1558 (Table S2), respectively. The two products were fused by overlapping PCR using primers #1652 and #1558 (Table S2), and the resulting DNA product was digested with KpnI and NotI and ligated into those sites of pSVP247.	This work

S1 Table. ***Continued.***

**Name**	**Description/Construction**[a]	**Source/Reference**
pMC72 (pCT144-2HA)	Derivative of pSVP247 encoding CT144-2HA expressed under the control of the *ct142* promoter. The promoter region of *ct142* and the *ct1144* gene were amplified by PCR from DNA of *C. trachomatis* L2/434 using primers #1652 and #1655 (Table S2), and #1656 and #1561 (Table S2), respectively. The two products were fused by overlapping PCR using primers #1652 and #1561 (Table S2). The resulting DNA product was digested with KpnI and NotI and ligated into those sites of pSVP247.	This work
pMC73 (pCT142-CT143-CT144-2HA)	Derivative of pSVP247 encoding CT142, CT143, and CT144-2HA, all expressed under the control of the *ct142* promoter. The promoter region of c*t142* and the *ct142-ct143-ct144* operon were amplified by PCR from DNA of *C. trachomatis* L2/434 using primers #1652 and #1561 (Table S2). The resulting DNA product was digested with KpnI and NotI and ligated into those sites of pSVP247.	This work
pSG1	Derivative of pEGFP-C1 encoding EGFP-CT142. *ct142* was amplified from genomic DNA of the *C. trachomatis* L2/434 strain using primers #622 and #623 (Table S2). The DNA product was digested with EcoRI and BamHI and ligated into those sites of pEGFP-C1.	
pSG2	Derivative of pEGFP-C1 encoding EGFP-CT143. *ct143* was amplified from genomic DNA of the *C. trachomatis* L2/434 strain using primers #624 and #617 (Table S2). The DNA product was digested with EcoRI and XhoI and ligated into those sites of pEGFP-C1.	

[a]Km^R, kanamycin resistance; Amp^R, ampicillin resistance.
