# Supplementary material for: The Chlamydia trachomatis type III secretion substrates CT142, CT143, and CT144 are secreted into the lumen of the inclusion
Source: PLoS One. 2017 Jun 16;12(6):e0178856. doi: 10.1371/journal.pone.0178856 (PMC5473537; doi:10.1371/journal.pone.0178856)
Supplement: S2 Table — (PDF) [file pone.0178856.s002.pdf]

S2 Table. DNA primers used in this work.

Code	Description	Sequence	Restriction enzyme[a]
#5	Construction of pSC1	GTACTCATGACGAATTCTATATCAGGTG	BspHI
#6	Construction of pSC1	GTACAAGCTTTTAAGCATAATCAGGAACATCATAC GGATACGCATAGTCCGGCACATCATACGGATATCC TACGGTATCAATCAGTGAGC	HindIII
#417	Construction of pMC47	GATCCTCGAGTTAATCTGCCTCCTTATAAGAAG	XhoI
#418	Construction of pCM33	GATCAAGCTTTTAAGGAACAACAGGTAGCCG	HindIII
#490	Construction of pCM33	CGCGGATCCAGTGATTCTGACAAAATTATT	BamHI
#491	Construction of pMC47	GATCGAATTCAGAAACCAGTATTTACAGGGG	EcoRI
#617	Construction of pSG2	GATCGAATTCTTAATCTGCCTCCTTATAAGAAG	EcoRI
#622	Construction of pSG1	GATCGAATTCTGATTCTGACAAAATTATTAATG	EcoRI
#623	Construction of pSG1	GATCGGATCCTTATCCTCCTATCTCTGGG	BamHI
#624	Construction of pSG2	GATCCTCGAGCTAAGAAACCAGTATTTACAGGG	XhoI
#1050	Construction of pMC56	GATCGAATTCAAGAAACCAGTATTTACAGG	EcoRI
#1051	Construction of pMC56	GATCCTGCAGTTAATCTGCCTCCTTATAAG	PstI
#1237	Transcription linkage (*ct141*-*ct142*)	GCAGGCGCTTCCACGGAAGG	-
#1238	Transcription linkage (*ct141*-*ct142*)	GGGAGTCTTTCTCCGGGC	-
#1239	Transcription linkage (*ct142*-*ct143*)	CTCAGGGACTACAGGCCTGC	-
#1240	Transcription linkage (*ct142*-*ct143*)	CCCTTTAACTAGTGCACC	-
#1241	Transcription linkage (*ct143*-*ct144*	CCTATGTATCAGAATCGG	-
#1242	Transcription linkage (*ct143*-*ct144*)	CCGGATAGCGCTGAACGC	-
#1243	Transcription linkage (*ct144*-*ct145*)	GGCATCTCCTGCGGCTCCCG	-

S2 Table. ***Continued.***

**Code**	**Description**	**Sequence**	**Restriction enzyme[a]**
#1244	Transcription linkage (*ct144*-*ct145*)	GCGCAAGAGAAATAGTCCC	-
#1255	5´RACE *ct142* Sp1	CCACCCCCATCGCTTCCC	-
#1256	5´RACE *ct142* Sp2	CGAGACGATTGATACACC	-
#1257	5´RACE *ct142* Sp3	GGCTACTTGATAGATGCCTGG	-
#1483	Construction of pSVP247	GATCGTCGACGTCTTAGGAGCTTTTTGCAATGC	SalI
#1486	Construction of pSVP247	GATCGCGGCCGCTATCCGTATGATGTGCCGGA	NotI
#1487	Construction of pSVP247	GATGTTCCTGATTATGCTTAAGGATGACATGTGATTCGCGTAGG	-
#1488	Construction of pSVP247	CCTACGCGAATCACATGTCATCCTTAAGCATAATCAGG	-
#1555	Construction of pMC70	GATCGCGGCCGCGTCCTCCTATCTCTGGGTATACG	NotI
#1558	Construction of pMC71	GATCGCGGCCGCGATCTGCCTCCTTATAAGAAGAACC	NotI
#1561	Construction of pMC72 and pMC73	GATCGCGGCCGCGAGGAACAACAGGTAGCCGAACC	NotI
#1652	Construction of pMC70, pMC71, pMC72, and pMC73	GATCGGTACCGGACCTCATAAAAATCCTATCAG	KpnI
#1653	Construction of pMC71	CCCTGTAAATACTGGTTTCTTCATTTAATTGTTTCCAAGTTTTTATTTTG	-
#1654	Construction of pMC71	CAAAATAAAAACTTGGAAACAATTAAATGAAGAAACCAGTATTTACAGGG	-
#1655	Construction of pMC72	GTATTATTATCTGGTGTTGTCATTTAATTGTTTCCAAGTTTTTATTTTG	-
#1656	Construction of pMC72	CAAAATAAAAACTTGGAAACAATTAAATGACAACACCAGATAATAATAC	-

[a]Restriction sites are underlined.
