# Supplementary material for: The Chlamydia trachomatis type III secretion substrates CT142, CT143, and CT144 are secreted into the lumen of the inclusion
Source: PLoS One. 2017 Jun 16;12(6):e0178856. doi: 10.1371/journal.pone.0178856 (PMC5473537; doi:10.1371/journal.pone.0178856)
Supplement: S3 Table — (PDF) [file pone.0178856.s003.pdf]

S3 Table. Identification of orthologues of *C. trachomatis* CT142 (CTL0397), CT143 (CTL0398), and CT144 (CTL0399) in other *Chlamydiae*[a]

	CTL0397 (CT142)		CTL0398 (CT143)		CTL0399 (CT144)	
	cover	Id	cover	Id	cover	Id
C. pneumoniae	96%	36%	96%<br>92%	35%<br>37%	99%	33%
C. muridarum	99%	71%	100%	74%	100%	68%
C. psittaci	83%	41%	93%	45%	98%	39%
C. abortus	99%	39%	93%	44%	98%	37%
C. caviae	96%	39%	93%	41%	96%	38%
C. pecorum	95%	34%	94%	37%	96%	40%
C. felis	95%	37%	97%	41%	98%	36%
C. avium	96%	42%	99%	39%	98%	36%
C. gallinacea	96%	41%	100%	42%	98%	37%
C. suis	100%	77%	100%	79%	99%	73%
Parachlamydiaceae	No Hits		No Hits		No Hits	
Waddliaceae	No Hits		No Hits		No Hits	
Simkaniaceae	No Hits		No Hits		No Hits	
Criblamydiaceae	No Hits		No Hits		No Hits	

[a]Orthologues of the *C. trachomatis* proteins CT142, CT143 and CT144 in other *Chlamydiae* were searched by PSI-BLAST [49]. An individual PSI-BLAST search was performed between each protein (using the corresponding amino acid sequence from *C. trachomatis* serovar L2 strain 434/Bu: CTL0397, CTL0398 and CTL0399, respectively) and a representative strain from each *Chlamydia* spp. (*C. pneumoniae* strain CWL029, *C. muridarum* strain Nigg., *C. psittaci* strain Mat116, *C. abortus* strain S26/3, *C. caviae* strain GPIC, *C. pecorum* PV3056/3, *C. felis* Fe/C-56, *C. avium* strain 10DC88, *C. gallinacean* 08-1274/3, *C. suis* strain MD56). Another individual PSI-BLAST search was performed between each protein and members of the other *Chlamydiae* families. The families *Rhabdochlamydiaceae, Piscichlamydeacea, Chlavichlamydiaceae* and *Parilichlamydiaceae* were excluded from our search because they were not included in the genome database. Cover, indicates % of coverage, and Id indicates % of identity.
