# Supplementary material for: The Chlamydia trachomatis type III secretion substrates CT142, CT143, and CT144 are secreted into the lumen of the inclusion
Source: PLoS One. 2017 Jun 16;12(6):e0178856. doi: 10.1371/journal.pone.0178856 (PMC5473537; doi:10.1371/journal.pone.0178856)
Supplement: S1 Fig — Details of plasmid construction are in S1 Table. The pSW2 plasmid backbone [27] is shown in black, the Escherichia coli origin of replication (ori) in yellow, the ampicillin resistance gene (bla) in blue, the multiple cloning site (MCS) in grey, and the double hemagglutinin (2HA) epitope tag and incD terminator in different tones of red. The DNA sequence of the MCS with unique restriction sites and the 2HA-encoding region are depicted in the box below the plasmid map. (PDF) [file pone.0178856.s004.pdf]

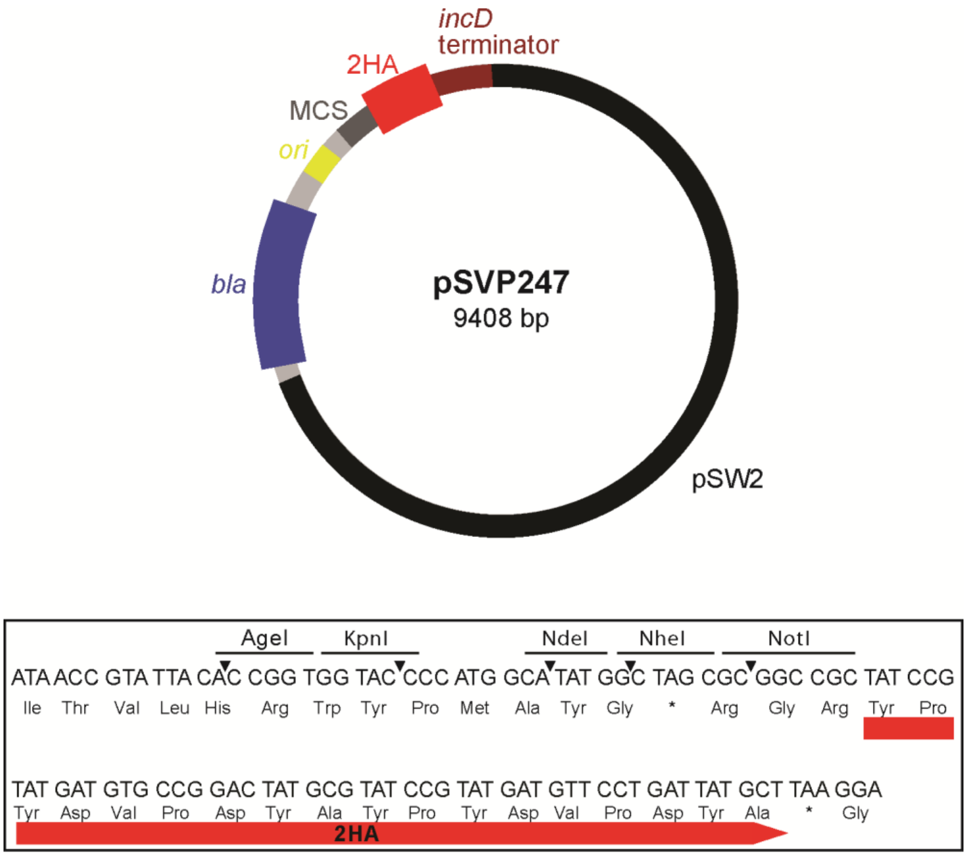

Features

pSW2 : 12...7173 bp

bla: 7422...8282 bp

ori: 8447...9035 bp

MCS: 9146...9183 bp

2xHA: 9184...9237 bp

incD terminator: 9241...9407 bp

S1 Fig. Map of plasmid pSVP247. Details of plasmid construction are in S1 Table. The pSW2 plasmid backbone [27] is shown in black, the *Escherichia coli* origin of replication (*ori*) in yellow, the ampicillin resistance gene (bla) in blue, the multiple cloning site (MCS) in grey, and the double hemagglutinin (2HA) epitope tag and *incD* terminator in different tones of red. The DNA sequence of the MCS with unique restriction sites and the 2HA-encoding region are depicted in the box below the plasmid map.
