# Supplementary material for: The Chlamydia trachomatis type III secretion substrates CT142, CT143, and CT144 are secreted into the lumen of the inclusion
Source: PLoS One. 2017 Jun 16;12(6):e0178856. doi: 10.1371/journal.pone.0178856 (PMC5473537; doi:10.1371/journal.pone.0178856)
Supplement: S2 Fig — Orthologues of ct142 are depicted in green, orthologues of ct143 are depicted in red, and orthologues of ct144 are depicted in pink. The syntenic organization of the three genes is illustrated in C. trachomatis serovar A (strain A/HAR-13), C. trachomatis serovar B (strain B/Jali20/OT), C. trachomatis serovar D (strain D/UW-3/CX) as well as in C. muridarum Nigg, C. abortus S26/3, C. caviae GPIC, C. felis Fe/C-56 and C. pneumoniae CWLO29. The initial image was obtained from the ChlamydiaeDB.org website (http://liferay.csb.univie.ac.at/portal/web/chlamydiaedb) and then adapted. (PDF) [file pone.0178856.s005.pdf]

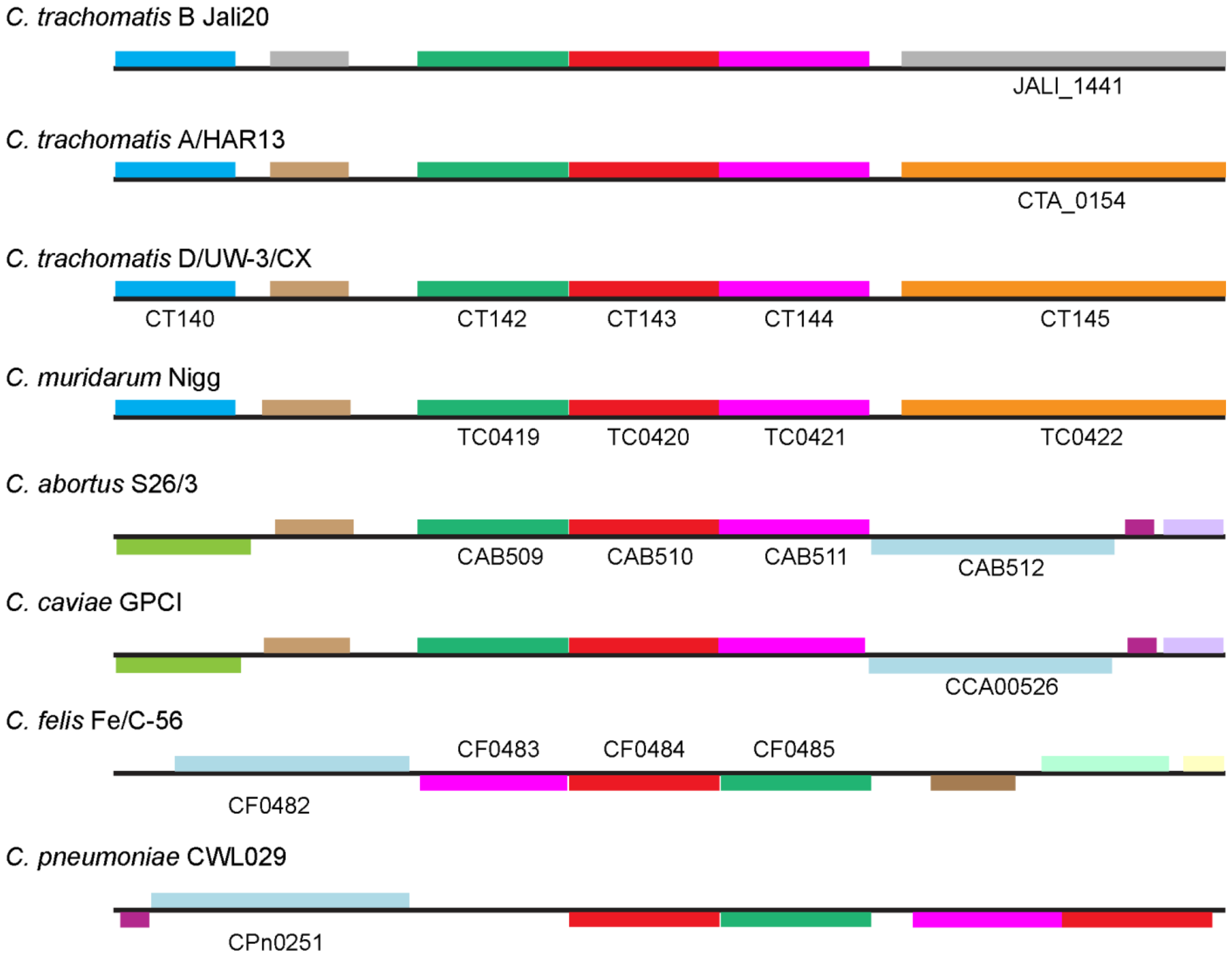

S2 Fig. Genetic organization of *ct142, ct143* and *ct144* orthologues in *Chlamydiaceae*. Orthologues of *ct142* are depicted in green, orthologues of *ct143* are depicted in red, and orthologues of *ct144* are depicted in pink. The syntenic organization of the three genes is illustrated in *C. trachomatis* serovar A (strain A/HAR-13), *C. trachomatis* serovar B (strain B/Jali20/OT), *C. trachomatis* serovar D (strain D/UW-3/CX) as well as in *C. muridarum* Nigg, *C. abortus* S26/3, *C. caviae* GPIC, *C. felis* Fe/C-56 and *C. pneumoniae* CWLO29. The initial image was obtained from the ChlamydiaeDB.org website (http://liferay.csb.univie.ac.at/portal/web/chlamydiaedb) and then adapted.
