# Supplementary material for: The Chlamydia trachomatis type III secretion substrates CT142, CT143, and CT144 are secreted into the lumen of the inclusion
Source: PLoS One. 2017 Jun 16;12(6):e0178856. doi: 10.1371/journal.pone.0178856 (PMC5473537; doi:10.1371/journal.pone.0178856)
Supplement: S3 Fig — HeLa cells were either left untransfected (NT) or transfected using jetPEI® (Polyplus-transfection) with plasmids encoding EGFP, EGFP-CT143 or EGFP-CT142, as indicated. Whole cell lysates were analyzed with antibodies against GFP, CT142 or CT143. Bands corresponding to EGFP-CT142 and EGFP-CT143 are indicated by an arrow. (PDF) [file pone.0178856.s006.pdf]

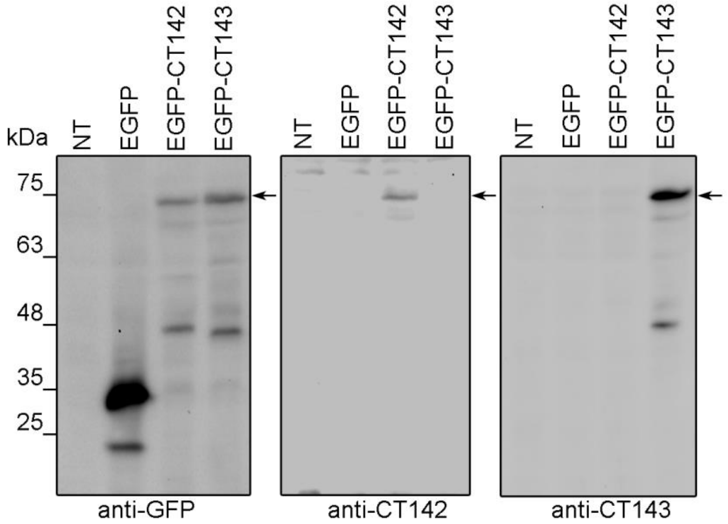

S3 Fig. Characterization of antibodies against CT142 and CT143. HeLa cells were either left untransfected (NT) or transfected using jetPEI® (Polyplus-transfection) with plasmids encoding EGFP, EGFP-CT143 or EGFP-CT142, as indicated. Whole cell lysates were analyzed with antibodies against GFP, CT142 or CT143. Bands corresponding to EGFP-CT142 and EGFP-CT143 are indicated by an arrow.
