# Supplementary material for: The Chlamydia trachomatis type III secretion substrates CT142, CT143, and CT144 are secreted into the lumen of the inclusion
Source: PLoS One. 2017 Jun 16;12(6):e0178856. doi: 10.1371/journal.pone.0178856 (PMC5473537; doi:10.1371/journal.pone.0178856)
Supplement: S4 Fig — (A) HeLa cells were left uninfected (UI) or infected for 30 h by C. trachomatis L2/434 or L2/434 carrying plasmids pCT142-2HA, pCT143-2HA, pCT144-2HA or pCT142-CT143-CT144-2HA. Whole cell lysates were analyzed by immunoblotting with antibodies against HA, CT142 or CT143 (as indicated), C. trachomatis Hsp60 (bacterial loading control) and tubulin (loading control for host cells). The arrows indicate the position of CT142, CT142-2HA, CT143 and CT143-2HA bands. (B) The bands corresponding to HA-tagged proteins in each lane of the anti-HA blot in Fig A in S4 Fig (and in other replicates) were quantified by densitometry relative to the corresponding bands of Hsp60 and tubulin using Fiji software [48]. The calculated CT142/Hsp60/tubulin values in the graph indicate mean ± standard error of the mean (SEM) from 4 independent experiments. P-values were calculated by a one way ANOVA and Tukey post hoc analysis and the values were not significantly different (P > 0.05) between the different data sets. (C and D) The bands corresponding to CT142 and CT142-2HA proteins (C), or the bands corresponding to CT143 and CT143-2HA proteins (D), in each lane of the anti-CT142 or CT143 blots, respectively, in Fig A in S4 Fig (and in other replicates) were quantified by densitometry relative to the corresponding bands of Hsp60 and tubulin using Fiji software [48]. The calculated HA/Hsp60/tubulin values in the graph indicate mean ± SEM from 4 independent experiments, relative to the values of CT142 (in C) or CT143 (in D) in cells infected by L2/434. P-values were calculated by a one way ANOVA and Dunett post hoc analysis (relative to the L2/434 data) and there were significant differences (P < 0.05) only for data corresponding to samples infected by L2/434 harbouring pCT142-2HA or pCT142-CT143-CT144-2HA (in C) or for data corresponding to samples infected by L2/434 harbouring pCT143-2HA. (PDF) [file pone.0178856.s007.pdf]

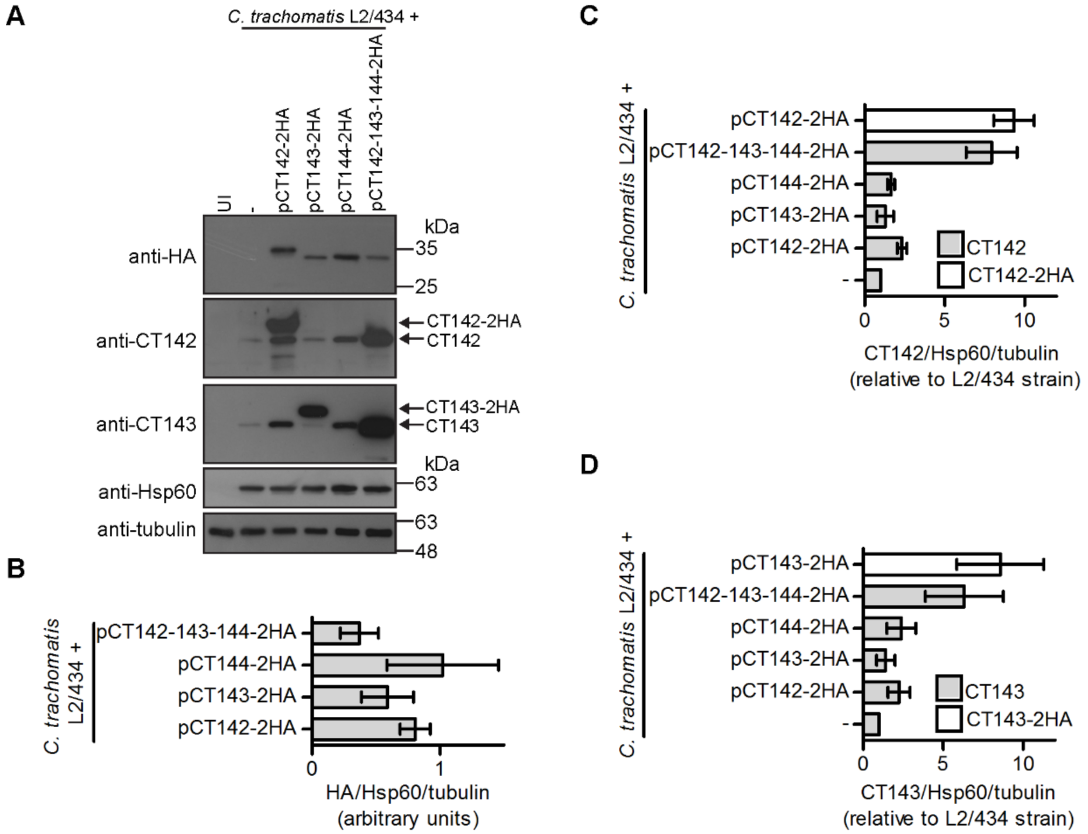

S4 Fig. Quantification of CT142, CT143, and CT144 proteins produced by *C. trachomatis* recombinant strains. (A) HeLa cells were left uninfected (UI) or infected for 30 h by *C. trachomatis* L2/434 or L2/434 carrying plasmids pCT142-2HA, pCT143-2HA, pCT144-2HA or pCT142-CT143-CT144-2HA. Whole cell lysates were analyzed by immunoblotting with antibodies against HA, CT142 or CT143 (as indicated), *C. trachomatis* Hsp60 (bacterial loading control) and tubulin (loading control for host cells). The arrows indicate the position of CT142, CT142-2HA, CT143 and CT143-2HA bands. (B) The bands corresponding to HA-tagged proteins in each lane of the anti-HA blot in Fig. S4A (and in other replicates) were quantified by densitometry relative to the corresponding bands of Hsp60 and tubulin using Fiji software [48]. The calculated CT142/Hsp60/tubulin values in the graph indicate mean ± standard error of the mean (SEM) from 4 independent experiments. P-values were calculated by a one way ANOVA and Tukey post hoc analysis and the values were not significantly different (P > 0.05) between the different data sets. (C and D) The bands corresponding to CT142 and CT142-2HA proteins (C), or the bands corresponding to CT143 and CT143-2HA proteins (D), in each lane of the anti-CT142 or CT143 blots, respectively, in Fig A in S4 Fig (and in other replicates) were quantified by densitometry relative to the corresponding bands of Hsp60 and tubulin using Fiji software [48]. The calculated HA/Hsp60/tubulin values in the graph indicate mean ± SEM from 4 independent experiments, relative to the values of CT142 (in C) or CT143 (in D) in cells infected by L2/434. P-values were calculated by a one way ANOVA and Dunett post hoc analysis (relative to the L2/434 data) and there were significant differences (P < 0.05) only for data corresponding to samples infected by L2/434 harbouring pCT142-2HA or pCT142-CT143-CT144-2HA (in C) or for data corresponding to samples infected by L2/434 harbouring pCT143-2HA.
